# The draft nuclear genome assembly of *Eucalyptus pauciflora*: a pipeline for comparing *de novo* assemblies

**DOI:** 10.1093/gigascience/giz160

**Published:** 2020-01-02

**Authors:** Weiwen Wang, Ashutosh Das, David Kainer, Miriam Schalamun, Alejandro Morales-Suarez, Benjamin Schwessinger, Robert Lanfear

**Affiliations:** 1 Research School of Biology, the Australian National University. 134 Linnaeus Way, Acton, Canberra, ACT, 2601, Australia; 2 Department of Genetics and Animal Breeding, Faculty of Veterinary Medicine, Chittagong Veterinary and Animal Sciences University. Khulshi, Chattogram, 4225, Bangladesh; 3 Institute of Applied Genetics and Cell Biology, University of Natural Resources and Life Sciences. Muthgasse 18, Vienna, 1190 Wien, Austria; 4 Department of Biological Sciences, Macquarie University.Building 6SR (E8B), 6 Science Rd, Sydney, NSW, 2109, Australia

**Keywords:** long-read assembly, nanopore sequencing, hybrid assembly, genome assessment, assembly comparison, *Eucalyptus pauciflora*, haplotig separation, genome polishing

## Abstract

**Background:**

*Eucalyptus pauciflora* (the snow gum) is a long-lived tree with high economic and ecological importance. Currently, little genomic information for *E. pauciflora* is available. Here, we sequentially assemble the genome of *Eucalyptus pauciflora* with different methods, and combine multiple existing and novel approaches to help to select the best genome assembly.

**Findings:**

We generated high coverage of long- (Nanopore, 174×) and short- (Illumina, 228×) read data from a single *E. pauciflora* individual and compared assemblies from 5 assemblers (Canu, SMARTdenovo, Flye, Marvel, and MaSuRCA) with different read lengths (1 and 35 kb minimum read length). A key component of our approach is to keep a randomly selected collection of ∼10% of both long and short reads separated from the assemblies to use as a validation set for assessing assemblies. Using this validation set along with a range of existing tools, we compared the assemblies in 8 ways: contig N50, BUSCO scores, LAI (long terminal repeat assembly index) scores, assembly ploidy, base-level error rate, CGAL (computing genome assembly likelihoods) scores, structural variation, and genome sequence similarity. Our result showed that MaSuRCA generated the best assembly, which is 594.87 Mb in size, with a contig N50 of 3.23 Mb, and an estimated error rate of ∼0.006 errors per base.

**Conclusions:**

We report a draft genome of *E. pauciflora*, which will be a valuable resource for further genomic studies of eucalypts. The approaches for assessing and comparing genomes should help in assessing and choosing among many potential genome assemblies from a single dataset.

## Data Description

### Introduction

**Figure 1:**
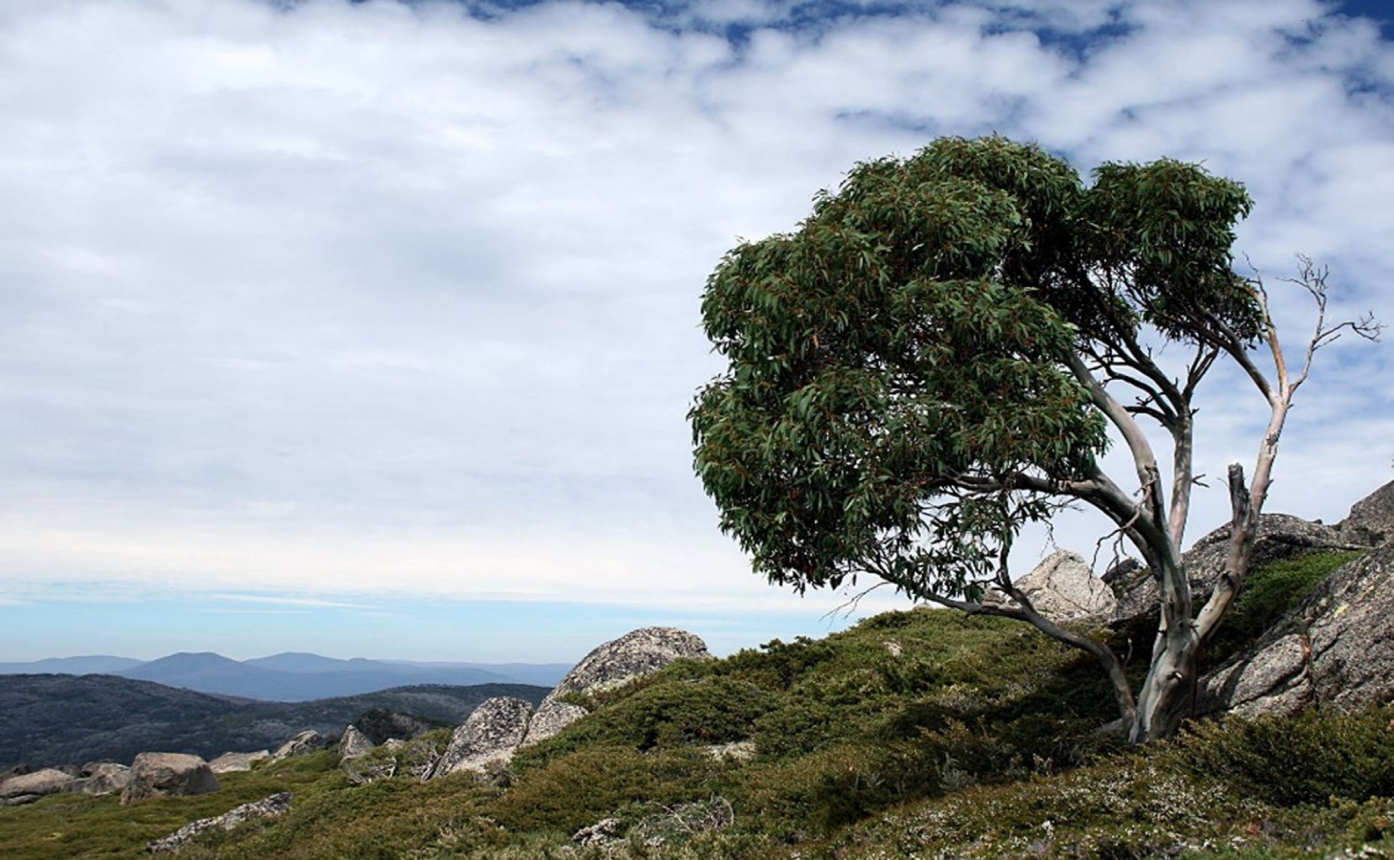
The *E. pauciflora* sequenced in this study. This *E. pauciflora* is located in Thredbo, Kosciuszko National Park, New South Wales, Australia (36 29.6597 N, 148 16.9788 E).

Eucalypts are widely distributed in Australia, including 3 genera, *Eucalyptus, Corymbia*, and *Angophora*, and have ∼800 species [1]. *Eucalyptus pauciflora* (NCBI:txid87676) (Fig. 1), also known as snow gum, is a highly variable eucalyptus species that inhabits diverse landscapes in southeastern Australia [1]. *E. pauciflora* can survive from close to sea level to up to the tree line of the Australian Alps, displaying the broadest altitudinal range in the eucalypt genera [2–4]. Owing to its wide distribution and drought and cold tolerance, *E. pauciflora* is used for carbon offset plantings, ecological restoration, honeybee food source, and also has medicinal uses [1, 5–11]. However, genomic resources for *E. pauciflora* are currently very limited: there exists a single chloroplast genome [12], 2 sets of microsatellite markers [13, 14], and 2 nuclear loci used for phylogenetics [15]. The assembly of *E. pauciflora* genome will assist in elucidating the genetic basis of drought and cold tolerance in *Eucalyptus*.

Across the ∼800 extant eucalypt species, there are only 2 genomes published: those for *Eucalyptus camaldulensis* and *Eucalyptus grandis* [16, 17]. Both of these genomes were sequenced with a combination of Sanger sequencing and short-read sequencing, and as a result both assemblies are somewhat fragmented. There are 81,246 scaffolds in the *E. camaldulensis* assembly [17]. While the *E. grandis* genome is highly contigous, assembled to chromosome level, it still has 4,941 unplaced scaffolds [16]. New technologies, such as third-generation long-read sequencing, have the potential to produce less fragmented assemblies at a fraction of the cost of previous methods. Nevertheless, many challenges still remain, not least of which is that different genome assembly software, and small changes to the parameters of a single piece of software, can produce substantially different assemblies. In light of this, methods for choosing the most accurate assembly from a set of possible assemblies have become increasingly important.

Two metrics are commonly used to assess and compare genome assemblies: contig N50 and BUSCO [18] (BUSCO, RRID:SCR_015008) scores. The contig N50 is the size of the contig such that ≥50% of the assembled nucleotides can be found in contigs of that size or larger. The N50 is a measure of genome contiguity, where a higher N50 suggests a genome that has been assembled into fewer and larger contigs. All else being equal, we should prefer genome assemblies with a larger N50, up to the point where the N50 is equal to the N50 of the chromosomes themselves. Perhaps because of this, the N50 is one of the most widely reported metrics in genome assembly. However, it is important to remember that the N50 measures contiguity, not accuracy. For example, N50 scores may be artificially inflated by incorrectly linking contigs [19, 20]. The BUSCO score estimates the proportion of highly conserved orthologous genes that are present in assemblies. The underlying assumption is that there exists a certain set of highly conserved single-copy genes, the vast majority of which we should expect to observe in single copies in any given haploid genome assembly. BUSCO scores provide a very useful measure of genome assembly completeness (a component of accuracy), and in principle we should prefer genome assemblies with BUSCO scores closer to 100%. One limitation of BUSCO scores is that they assess only a very small proportion of the genome, typically ∼1,000 highly conserved genes that represent <1% of the total genome. Furthermore, by their nature these protein-coding regions of the genome tend to be among the easiest to assemble because they are usually single-copy regions. Hence, assemblies can have very similar BUSCO scores even if they differ considerably in their assembly of the non-BUSCO genomic regions, which means that it is sometimes difficult to use BUSCO scores to distinguish among competing assemblies [21]. In this study, we complement these commonly used measures with a range of other metrics to assess and compare genome assemblies, and we use these measures to choose the best draft assembly of *E. pauciflora*.

One measure we propose is the assembly ploidy: the proportion of the genome that is represented by haploid contigs. One important problem in genome assembly is that we commonly represent the genome of diploid (or polyploid) organisms as a haploid sequence. Traditionally, genome projects would alleviate this problem by sequencing highly inbred individuals [22, 23], thus reducing the discrepancy between the diploid individual and the haploid representation. However, as genome assembly has become more commonplace, we often want to assemble the genomes of highly heterozygous individuals. For example, heterozygosity in *Eucalyptus* is ∼1% [24] and varies substantially along the genome [16]. The consequence of this is that regions of low heterozygosity tend to be assembled into a single collapsed haploid sequence, whereas regions of high heterozygosity tend to be assembled into 2 haplotypes of the same region, which are usually labelled the “primary contig” (referring to the longer of the 2 contigs) and the “haplotig” (referring to the shorter of the 2 contigs) [25]. Although there has been some progresses in estimating truly diploid assemblies [25, 26], most assemblers still produce primary contigs and haplotigs without labelling them as such [27, 28]. Crucially, unidentified haplotigs may cause issues in the downstream analyses, because many analyses assume that we have a haploid representation of the genome. Because of this, we propose a novel and simple (but imperfect) metric to measure the assembly ploidy, which is simply the ratio of the assembly size to the estimated haploid genome size. If the aim is to produce a haploid representation of a genome, then an assembly ploidy of 1 is preferable (i.e., the assembly size should be equal to the estimated haploid genome size). If the aim is to produce a diploid representation of a genome, then an assembly ploidy of 2 is preferable (i.e., the assembly size should be double the estimated haploid genome size). One limitation of this metric is that it is sensitive to errors in the estimation of haploid genome size, and it is also sensitive to errors in genome assembly (e.g., highly incomplete assemblies) that might affect the numerator. Nevertheless, in combination with other measures, we show below that the assembly ploidy provides a useful metric for comparing genome assemblies.

We also apply a suite of measures designed to provide a genome-wide assessment of contiguity and accuracy that can complement the widely used contig N50 and BUSCO scores. The advantages of these measures lie in the fact that they assess more of the genome than BUSCO scores, although each also has its limitations. Several tools have been developed to evaluate the quality of assemblies given an alignment of sequencing reads to the assembly, including FRCbam (FRCbam, RRID:SCR_005189) [29], REAPR (Recognition of Errors in Assemblies using Paired Reads, RRID:SCR_017625) [30], and CGAL (Computing Genome Assembly Likelihoods, RRID:SCR_017624) [20]. All of these tools require read alignment information. FRCbam first computes a series of features with the alignment information and then creates feature response curves that can be used to assess and compare assemblies. REAPR uses the read alignment to identify possibly misassembled regions and to give a score for the accuracy of each base in the genome. CGAL provides the likelihood of an assembly, calculated from a model that accounts for errors in reads, read coverage across the assembly, and the proportion of reads that do not contribute to the assembly. Of these 3 related tools, we use CGAL in this study because it provides a single likelihood score for each assembly, such that a higher likelihood from CGAL suggests that a genome assembly is a better representation of the truth, making it very simple to compare multiple assemblies. The second measure we used is the long-terminal repeat (LTR) assembly index, or LAI (LTR_retriever, RRID:SCR_017623) [21]. The LAI score is the proportion of LTR sequences in the genome that are intact, and is independent of genome size and repeat content. In general, a higher LAI score suggests a more contiguous and complete assembly [21]. The third measure we use is the base-level error rate evaluated by re-mapping independent sets of long and short validation reads (∼10% of all reads, randomly selected) to the assembly. Previous studies have evaluated the base-level error rate by re-mapping all reads to the assembly [31, 32]. Here, we use validation reads that are not involved in the assembly, in order to avoid any possible biases introduced by validating an assembly with the same data that were used to produce it. For a perfect assembly in which the ploidy of the entire assembly matches the ploidy of the individual, a lower base-level error rate is preferable, with a theoretical minimum of the error rate of the sequencing technology (e.g., ∼0.3% for raw Illumina reads [33] and ∼10–15% for raw Nanopore reads [34, 35]). For a haploid representation of a diploid assembly, the minimum possible base-level error rate will be higher because by necessity a haploid representation of a heterozygous site will not match approximately half of the reads. In this case, the theoretical minimum base-level error rate is the sum of the error rate of the sequencing technology and half of the heterozygosity. The fourth measure we use is the number of structural variants detected when re-mapping our long validation reads to assemblies. As with the base-level error rate, if the ploidy of the assembly matches the ploidy of the individual, then the theoretical minimum of this metric is the structural error rate introduced into sequencing reads by the sequencing technology. For a haploid representation of a diploid genome, the theoretical minimum is the sum of the error rate of the technology plus half of the structural heterozygosity. These 2 quantities are rarely known, but nevertheless, a very high structural error rate of validation reads mapped to a haploid assembly may indicate cases in which the assembly has a large proportion of incorrectly linked contigs. The final measure is the genome sequence similarity of each assembly when compared to all other assemblies. This measure does not provide any information relative to an underlying truth, but it may help to identify significant differences between otherwise plausible genome assemblies that can aid in choosing the best assembly. The selection of the best assembly should consider all measures together.

Here, we used long and short reads to create a draft haploid assembly of the *E. pauciflora* genome. We use the metrics we describe above to compare a range of assemblies from a range of different assemblers. We performed different assemblies with long-read–only assemblers (Canu [Canu, RRID:SCR_015880] [36], SMARTdenovo [SMARTdenovo, RRID:SCR_017622] [37], Flye [Flye, RRID:SCR_017016] [38], and Marvel [Marvel, RRID:SCR_017621] [39]) and a hybrid assembler, MaSuRCA (MaSuRCA, RRID:SCR_010691) [40], using long-read datasets with different minimum read lengths in each case (1 and 35 kb).

### Sample collection, DNA sequencing, and quality control

We collected leaves from the single *E. pauciflora* tree near Thredbo, Kosciuszko National Park, New South Wales, Australia (36 29.6597 N, 148 16.9788 E) in March 2016 (for Illumina sequencing) and June 2017 (for MinION sequencing). We stored leaves at 4°C when transporting them to the laboratory.

For long-read sequencing, we extracted high molecular weight genomic DNA from leaves following a protocol optimized for *Eucalyptus* nanopore sequencing [41]. We prepared Oxford Nanopore Technologies 1D ligation libraries according to the manufacturer's protocol, SQK-LSK108 (Oxford Nanopore Technologies Ltd, Oxford, United kingdom), and sequenced the reads using MinKNOW v1.7.3 with R9.5 flowcells on a MinION sequencer. We performed base calling with Albacore v2.0.2 (Albacore, RRID:SCR_015897). This resulted in 12,584,100 raw long reads (106.96 Gb) with average read length of 8.5 kb. We removed adapters from long reads with Porechop v0.2.1 (Porechop, RRID:SCR_016967) [42]. Next, we trimmed bases with quality <10 on both ends of the reads using NanoFilt v2.0.0 (NanoFilt, RRID:SCR_016966) [43] and discarded reads shorter than 1 kb after trimming. This recovered 96.66 Gb of long-read data comprising 7,711,141 filtered reads with an average read length of 12.53 kb (minimum 1 kb and maximum ∼150 kb). Given an estimated genome size of 500 Mb (see below), this represents a coverage of 193×.

For short-read sequencing, we extracted genomic DNA from freeze-dried leaves using a CTAB protocol [44] followed by purification with a Zymo kit (Zymo Research Corp, Irvine, California, United States). We constructed TruSeq Nano libraries with an insert size of 400 bp using protocol provided by Illumina, then sequenced the reads (paired-end 150 bp) using an Illumina Hiseq2500 platform (Illumina Inc., San Diego, California, United States). This Illumina sequencing generated 506,840,789 paired raw reads (152.05 Gb). We used BBDuk v37.31 (BBmap, RRID:SCR_016965) [45] to remove adapters and to trim both sides of raw short reads for which quality was <30. We discarded filtered reads with length <50 bp. Approximately 122.69 Gb short-read data containing 414,697,585 paired reads were left, representing 246× coverage with an estimated genome size of 500 Mb (see below).

### Genome size estimation

We used GenomeScope (GenomeScope, RRID:SCR_017014) [46] and SGA-preqc (SGA, RRID:SCR_001982) [47] to estimate the *E. pauciflora* genome size. We first generated a 32-mer distribution using Jellyfish v1.1.12 (Jellyfish, RRID:SCR_005491) [48] from all of our short reads, then ran GenomeScope using this 32-mer distribution with a maximum *k*-mer coverage of 1,000×. This gave a genome size estimate of 408.16 Mb (Fig. S1), which is lower than expected for other *Eucalyptus* species [16, 17]. However, it is known that genomic repeats can lead to underestimation of genome sizes from uncorrected *k-*mer distributions [49], and the *Eucalyptus* genome is repeat-rich, e.g., ∼50% of genome was annotated as repeats in *E. grandis* [16], suggesting that 408.16 Mb may be a significant underestimate of the genome size. Also, GenomeScope suggests that the heterozygosity of *E. pauciflora* is 1.5%. SGA-preqc estimates genome size from *k*-mer distributions that are corrected to attempt to better account for repeat content; in line with this, SGA-preqc gave a genome size estimate of 529.40 Mb. Because of this, we expect that the SGA-preqc genome size is likely to be more accurate, and in what follows we assume that the *E. pauciflora* genome size is ∼500 Mb. This suggests that the *E. pauciflora* genome may be ∼30% smaller than that of the other 2 sequenced *Eucalyptus* species, *E. grandis* (691.43 Mb) [16] and *E. camaldulensis* (654.92 Mb) [17]. However, the genome sizes of *E. grandis* and *E. camaldulensis* may be overestimated owing to the assembly and scaffolding of both haplotypes at high-heterozygosity regions.

### Creation of assembly and validation datasets

We separated our long-read and short-read data into assembly and validation datasets by randomly assigning the trimmed and filtered reads into the 2 datasets with custom scripts [50]. The assembly dataset comprised 86.98 Gb of long-read data (174× coverage) and 114.10 Gb of short-read data (228× coverage). The validation dataset comprised 9.67 Gb of long-read data (19× coverage, 10% of total long reads) and 8.59 Gb of short-read data (17× coverage, 7% of total short reads).

### Genome assembly

Here, we compared 7 long-read–only assemblies and 2 hybrid assemblies. For each combination of data and genome assembler, we followed the same genome assembly pipeline. We first used the assembler to produce an initial assembly. Following this, we identified and removed contigs from contaminant sequences and then polished the resulting assembly. We then identified and removed haplotigs from the assembly. Each assembly was re-polished after haplotig removal. To select the best assembly, we calculated the contig N50 with Quast v4.6.0 (QUAST, RRID:SCR_001228) [19], BUSCO scores with BUSCO v3.0.2, and LAI scores using the LTR_retriever pipeline [51]. After mapping the long and short validation reads to the final assemblies (using Ngmlr v0.2.6 [Ngmlr, RRID:SCR_017620] [52] for the former and Bowtie2 v2.3.4.1 [Bowtie2, RRID:SCR_016368] [53] for the latter), we calculated the base-level error rate using QualiMap v2.2.1 (QualiMap, RRID:SCR_001209) [54], the structural variant error rate using Sniffles v1.0.8 (Sniffles, RRID:SCR_017619) [52], and CGAL scores using CGAL. Finally, we performed whole-genome alignment between different assemblies with the NUCmer module of MUMmer v4.0.0beta2 (MUMmerGPU, RRID:SCR_001200) [55].

Oxford Nanopore reads tend to have error rates of ∼10–15%, which can make assembly of uncorrected reads very challenging. To alleviate this, we first corrected the long-reads assembly dataset with Canu v1.6 with default parameters except for setting corMinCoverage to 8, meaning that read correction would only be applied where ≥8 reads overlapped. We deemed this reasonable given the very high coverage of our data (174×). We then put the corrected long-read datasets into 2 sets for assembly. The first dataset contained all corrected long reads, such that the minimum read length was 1 kb (174× of coverage). The second dataset contained all corrected reads longer than 35 kb (∼40× of coverage). We refer to these datasets as the 1 kb and the 35 kb datasets, respectively.

We first compared the performance of using corrected and uncorrected long reads to assemble the genome with 2 efficient assemblers, Flye v2.3.5 and wtdbg2 v2.5 (WTDBG, RRID:SCR_017225) [56] (Supplementary Results). The results showed clearly that corrected long reads produced better assemblies than uncorrected long reads using Flye, while the differences with wtdbg2 were less pronounced (Table S1). Nevertheless, the Flye assemblies with corrected reads were the best overall, so we therefore decided to use corrected long reads for the rest of the assemblies in the study.

We attempted 8 long-read–only assemblies and 2 hybrid assemblies. Assemblies solely with long-read data were performed on corrected reads of 2 read lengths (1 and 35 kb) using 4 long-read assemblers: Canu v1.6 and v1.7, SMARTdenovo, Flye v2.3.5, and Marvel v1.0. The Marvel assembly with 1 kb dataset was not feasible because it required more disk space than we had available, resulting in 7 successful long-read–only assemblies. We used MaSuRCA v3.2.6 to perform hybrid assemblies with both read length datasets (1 and 35 kb) each combined with the short-read dataset. In what follows, we refer to these assemblies as Canu_1 kb, Canu_35 kb, SMARTdenovo_1 kb, SMARTdenovo_35 kb, Flye_1 kb, Flye_35 kb, Marvel_35 kb, MaSuRCA_1 kb, and MaSuRCA_35 kb. In general, we used default settings in all assemblers, and an estimated genome size of 500 Mb where this setting was required. For Canu assemblies, the 1 kb dataset was assembled using Canu v1.6, whereas the 35 kb dataset was assembled using Canu v1.7. We did not repeat the Canu_1 kb assembly after Canu v1.7 was released because we no longer had sufficient computational resources. For the Flye assembler, we used the “nano-cor” parameter, which accounts for the use of corrected nanopore reads. The chloroplast genome and mitochondrial genome were removed from each assembly by searching for the relevant contigs using BLASTN v2.7.1+ (BLASTN, RRID:SCR_001598) [57] with an E-value cutoff of ≤1 × 10^−20^. For each assembly, we recorded the runtime in CPU hours, the raw assembly length, and the N50 (Table 1).

**Table 1:** Raw (before polish and haplotig removal) assembly statistics

Assembly	Long-read^^^	Short-read	Assembler	Assembly time (CPU hours)*	Length (bp)	contigs	Largest contig (bp)	N50 (bp)	L50	GC (%)	Ns (%)
Canu_1kb	≥1 kb (∼174×)	X	Canu	∼300,000	871,577,052	2,867	7,123,373	629,835	259	39.18	0
Canu_35kb	≥35 kb (∼40×)	X	Canu	∼50,000	825,916,527	2,550	10,153,603	962,598	158	39.18	0
SMARTdenovo_1kb	≥1 kb (∼174×)	X	SMARTdenovo	∼8,000	610,858,639	729	6,287,341	1,711,661	107	39.29	0
SMARTdenovo_35kb	≥35 kb (∼40×)	X	SMARTdenovo	∼4,000	586,903,502	704	9,494,401	1,868,532	91	39.27	0
Flye_1kb	≥1 kb (∼174×)	X	Flye	∼700	596,007,484	5,930	2,755,662	255,434	652	39.12	0
Flye_35kb	≥35 kb (∼40×)	X	Flye	∼500	561,349,738	4,145	2,407,003	352,050	448	39.17	0
Marvel_35kb	≥35 kb (∼40×)	X	Marvel	∼28,000	649,061,435	1,181	6,453,759	795,971	182	39.07	0
MaSuRCA_1kb	≥1,kb (∼174×)	∼228×	MaSuRCA	∼23,000	778,288,575	1,311	12,224,271	1,885,174	95	39.35	0.04
MaSuRCA_35kb	≥35 kb (∼40×)	∼228×	MaSuRCA	∼21,000	773,035,614	1,703	8,684,546	1,304,720	146	39.39	0.09

^ All long reads were corrected by Canu before assembly. The Canu correction step took ∼200,000 CPU hours, which has not been included in the assembly runtime.

* With ∼1 TB of RAM.

### Contamination detection

Following initial assembly, we used Blobtools v1.0.1 (Blobtools, RRID:SCR_017618) [58] to assess contamination in each genome assembly. To do this, we first generated a hit file for each assembly by searching all contigs against the NCBI non-redundant nucleotide database using BLASTN v2.7.1+ (E-value ≤ 1 × 10^−20^). We then analysed the hit file for each assembly using Blobtools, which provides taxonomic annotations and other diagnostic plots to detect contamination in raw genome assemblies. The top hit was streptophyta phylum, comprising 99.72–100% of the hits in different assemblies (Fig. S2), indicating that there was no potential contamination from a non-plant origin in each raw assembly.

### Genome polishing

We polished each initial genome assembly to improve its accuracy. For the Canu, SMARTdenovo, Flye, and Marvel assemblies (i.e., those built from long reads only), we polished first with Racon v0.5 [59] using Ngmlr using the long-read assembly dataset, and then with Pilon v1.22 (Pilon, RRID:SCR_014731) [60] using Bowtie2 with the short-read assembly dataset. For the MaSuRCA assemblies, we polished only with Pilon because MaSuRCA is a hybrid assembler, and using error-prone long reads to polish hybrid assemblies tends to induce more errors rather than remove them (Table S2).

We ran each polishing algorithm for multiple iterations until the accuracy of the resulting assembly stopped improving or improved only slightly. We assessed the improvements using BUSCO scores and the base-level error rate by re-mapping validation long and short reads to each assembly (mapped as above). We evaluated the BUSCO scores using BUSCO with the embryophyta_odb9 lineage (1,440 genes in total). Polishing with Racon took between 2 and 12 iterations, and with Pilon between 3 and 10 iterations (Table S2).

Polishing with both Racon and Pilon significantly improved all of the raw genome assemblies, measured with base-level errors in long and short reads, and with BUSCO scores (Table S2). Polishing with Racon improved long-read base-level accuracy by up to 0.83% (in the Marvel_35 kb assembly), short-read base-level accuracy by up to 1.51% (also in the Marvel_35 kb assembly), and the BUSCO completeness scores by up to 30.76% (in the Flye_35 kb assembly). Polishing with Pilon further improved the long-read base-level accuracy by up to 0.40% (in the Marvel_35 kb assembly), the short-read base-level accuracy by up to 1.41% (in the Flye_35 kb assembly), and the BUSCO completeness scores by up to 24.44% (in the Flye_1 kb assembly).

### Assembly ploidy and haplotig removal

**Figure 2:**
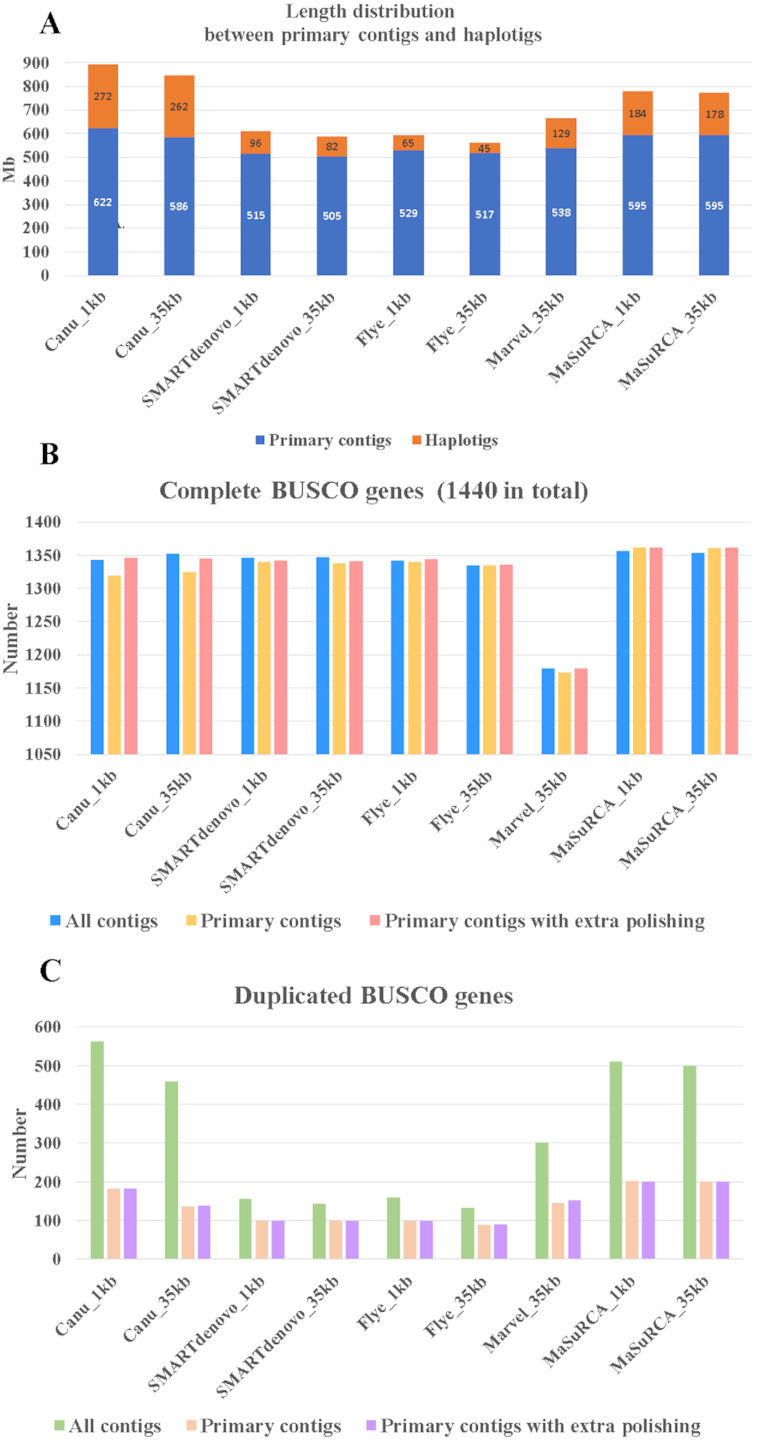
**A**. The length of primary contigs and haplotigs between different assemblies. **B**. The comparison of complete BUSCO genes (1,440 in total) between different primary contigs. **C**. The comparison of duplicated BUSCO genes between different primary contigs.

Comparison of the polished genome assemblies revealed large variation in assembly size (Table 2). We calculated the assembly ploidy of each assembly as described above, assuming a genome size of 500 Mb. The assembly ploidy ranges from 1.12 (Flye_35 kb assembly) to 1.79 (Canu_1 kb assembly) (Table 2), suggesting that the Canu_1 kb assembly is close to a diploid assembly (i.e., ∼80% of the genome is represented by 2 contigs) and that the Flye_35 kb assembly is close to a haploid assembly (i.e., only ∼12% of the genome is represented by 2 contigs). To attempt to produce haploid representations of the genome from all assemblies, we used Purge Haplotigs (Purge_haplotigs, RRID:SCR_017616) [28] and a custom pipeline, which we call gene conservation informed contig alignment (GCICA, RRID:SCR_017617) (script available on Github from [61]), to find and remove haplotigs from all the assemblies (Fig. 2A).

**Table 2:** Assembly size and assembly ploidy during polishing and haplotig removal

	Stage 1	Assembly ploidy	Stage 2	Assembly ploidy	Stage 3	Assembly ploidy	Stage 4	Assembly ploidy	Stage 5	Assembly ploidy
Canu_1kb	871,577,052	1.74	893,781,515	1.79	645,703,255	1.29	622,473,836	1.24	622,218,742	1.24
Canu_35kb	825,916,527	1.65	847,395,928	1.69	605,520,689	1.21	586,032,599	1.17	585,785,283	1.17
SMARTdenovo_1kb	599,580,691	1.20	610,858,639	1.22	514,822,476	1.03	514,822,476	1.03	514,714,831	1.03
SMARTdenovo_35kb	575,805,356	1.15	586,903,502	1.17	504,644,753	1.01	504,644,753	1.01	504,515,539	1.01
Flye_1kb	596,007,484	1.19	593,219,654	1.19	529,107,244	1.06	528,619,533	1.06	528,563,896	1.06
Flye_35kb	561,349,738	1.12	561,597,192	1.12	517,329,093	1.03	517,061,277	1.03	516,992,152	1.03
Marvel_35kb	649,061,435	1.30	666,317,308	1.33	547,630,224	1.10	537,813,575	1.08	537,615,613	1.08
MaSuRCA_1kb	778,288,575	1.56	778,307,850	1.56	608,764,671	1.22	594,680,200	1.19	594,528,099	1.19
MaSuRCA_35kb	773,035,614	1.55	773,071,231	1.55	608,629,204	1.22	595,020,257	1.19	594,871,467	1.19

Stage 1: raw assembly size (bp) before polishing. Stage 2: assembly size (bp) after polishing. Stage 3: assembly size (bp) after Purge Haplotigs. Stage 4: assembly size (bp) after Purge Haplotigs and GCICA (bp). Stage 5: assembly size (bp) after Purge Haplotigs and GCICA and extra polishing.

Purge Haplotigs assigns contigs to primary contigs and haplotigs depending on both coverage information generated by long-read mapping and pairwise alignments of all contigs. To run Purge Haplotigs, we first mapped the long-read assembly dataset to each polished assembly using Ngmlr and then separated the contigs into primary contigs and haplotigs with default settings. A total of 8–29% of each genome assembly (after polishing) was annotated as haplotigs, and removing these haplotigs reduced the assembly ploidy from 1.12–1.79 to 1.01–1.24 (Table 2).

The high assembly ploidy for some assemblies after running Purge Haplotigs suggested that these assemblies retained haplotigs that covered up to 29% of the genome. We therefore further filtered possible haplotigs using a custom approach, GCICA. If a pair of contigs comprise a primary contig and a haplotig, we would expect most of the regions of the haplotig to be very similar to that of the primary contig. To find putative pairs of primary contigs and haplotigs, we therefore looked for pairs of contigs with similar gene content, and then examined these pairs in more detail. To do this, we first mapped the nucleotide sequences of all *E. grandis* genes to all contigs in an assembly using BLASTN (E-value ≤ 1 × 10^−5^). If >70% of mapped markers in a contig could also be mapped to another contig, and ≥80% of sequence of the smaller contig could be aligned to the other contig (detecting with NUCmer module of MUMmer), we considered these 2 contigs as a putative primary contig and haplotig pair. We then examined the alignments of all such pairs by eye and removed any pairs in which the smaller contig appeared to be completely contained within the larger, i.e., in which the smaller contig was an unambiguous haplotig. This process identified a further ∼0–2% of each assembly as haplotigs (Table 2).

Following removal of haplotigs, we re-evaluated each assembly using BUSCO scores (Fig. 2B and C). We noted that, depending on the genome assembly, the number of complete BUSCO genes sometimes decreased and sometimes increased slightly after removal of haplotigs (Fig. 2B). We hypothesized that BUSCO scores could decrease either because haplotig removal mistakenly removed a contig that was not a haplotig or because haplotig removal correctly removed a haplotig that contained a more conserved representation of a BUSCO gene. BUSCO scores could increase because they are based on E-value scores of alignments, which may be affected by the total length of the assembly. To attempt to alleviate some of these potential issues, we re-polished all of the genome assemblies with multiple rounds of Pilon using the short-read assembly dataset, as above. BUSCO scores recovered across all assemblies with additional Pilon polishing (Fig. 2B). As expected, the number of duplicated BUSCO genes decreased substantially (∼50–70%) after haplotigs were removed from the assemblies and this did not change substantially after additional polishing (Fig. 2C and Table S3). Together, these results suggest that our haplotig removal pipelines largely succeeded in removing haplotigs, although some haplotigs likely remain if the true genome size is ∼500 Mb (Fig. 2A).

### Assessment of assembly quality with 8 measures

**Figure 3:**
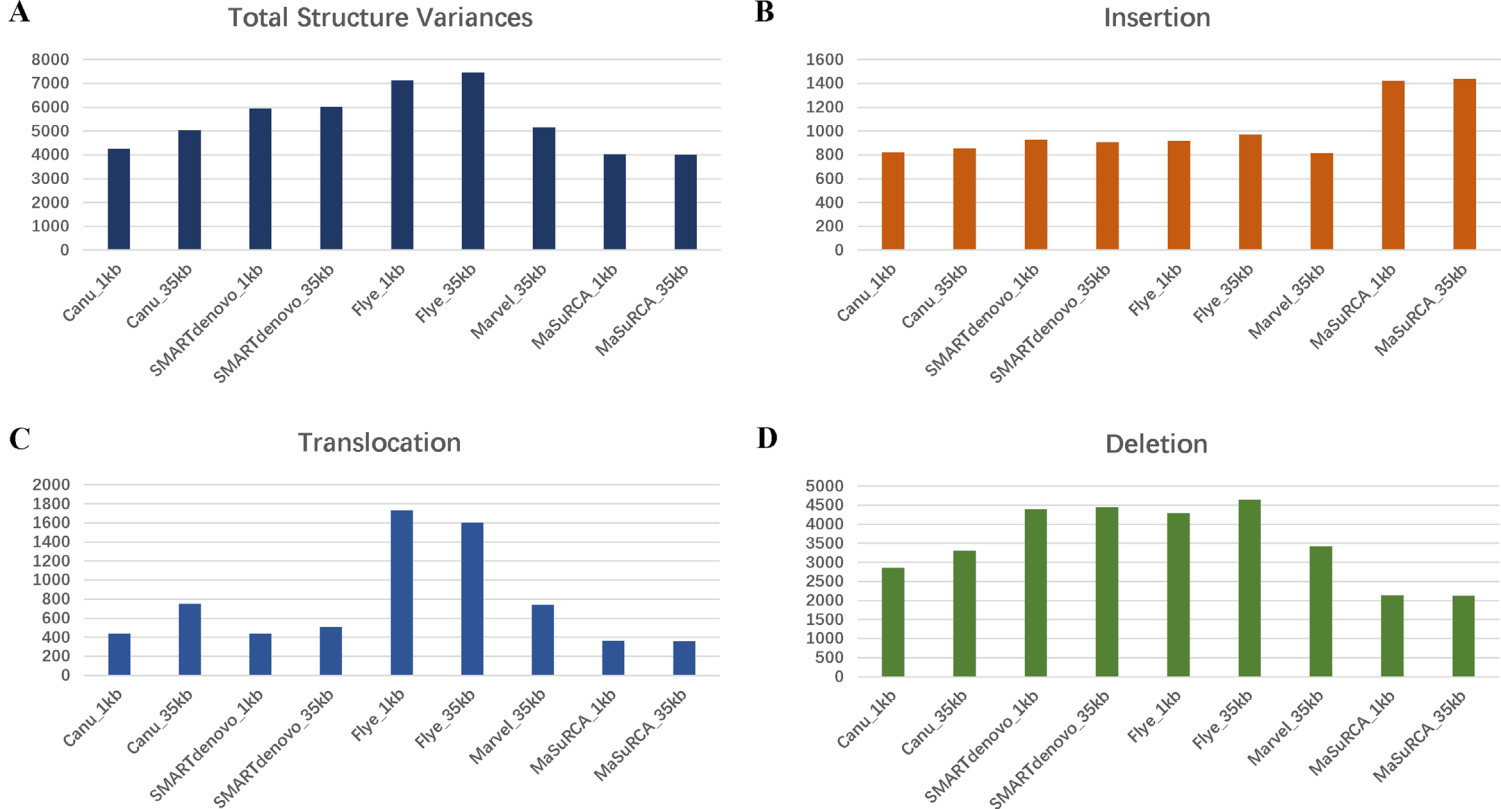
Structural variation analysis of different assembly primary contigs. Each variant was supported by ≥10 long reads. **A**. The total event of each structural variant of each assembly. **B**. The insertion event of each assembly. **C**. The translocation event of each assembly. **D**. The deletion event of each assembly.

**Figure 4:**
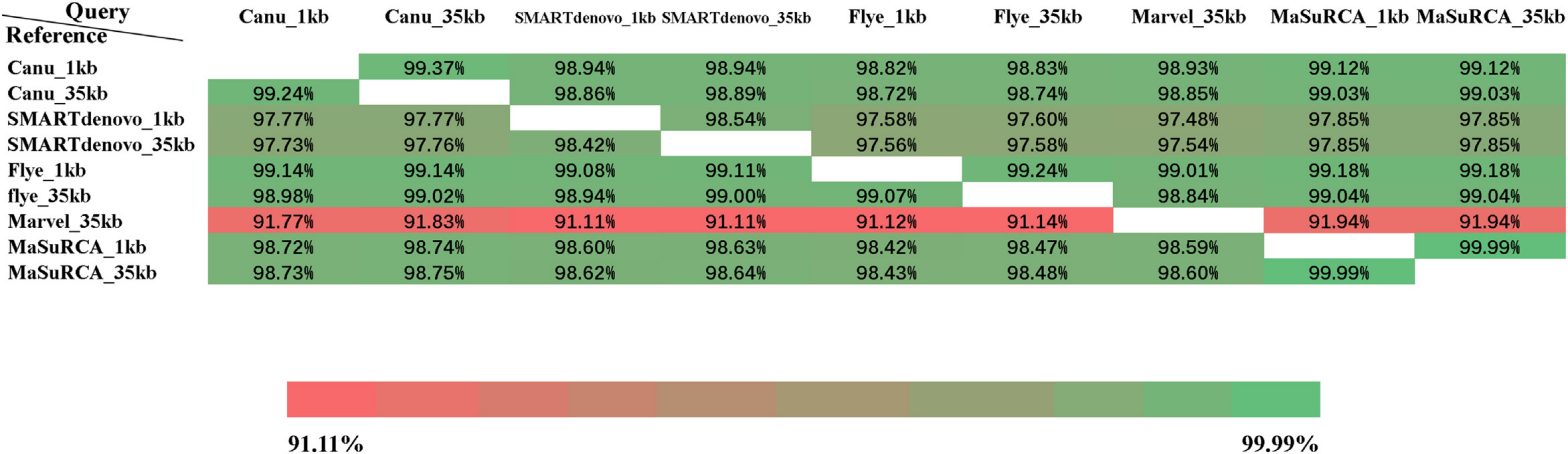
The sequence coverage of whole-genome alignment among different assemblies. The sequence coverage was calculated by the length of aligned reference sequence/the total length of reference genome.

After haplotig removal and polishing, we considered the primary contigs of each assembly as the final assembly, and evaluated each of the final assemblies by means of the 8 statistics we describe above: contig N50, BUSCO score, LAI score, assembly ploidy, base-level error rate, CGAL score, structural variation, and genome sequence similarity (Table 3 and Figs 3 and 4).

**Table 3:** The comparison of final assemblies

Assembly	Length (bp)	Contig No.	Contig N50 (bp)	BUSCO score (1,440 genes in total)						LAI score	Assembly ploidy	Short-read mapping		Long-read mapping		CGAL score	Structural variants
				Complete genes		Duplicated genes		Fragmented genes				Mapping rate	Error rate	Mapping rate	Error rate		
Canu_1kb	622,218,742	895	1,502,325	1,346	93.47%	183	12.71%	23	1.60%	7.04	1.24	96.02%	0.0061	91.73%	0.1661	−1.959E+06	4,243
Canu_35kb	585,785,283	655	2,258,674	1,345	93.40%	138	9.58%	29	2.01%	5.34	1.17	95.52%	0.0066	92.64%	0.1677	−2.226E+06	5,043
SMARTdenovo_1kb	514,714,831	364	2,092,790	1,342	93.19%	100	6.94%	27	1.88%	7.02	1.03	**98.42%**	0.0080	92.38%	0.1678	−4.275E+06	5,940
SMARTdenovo_35kb	504,515,539	370	2,178,079	1,341	93.13%	100	6.94%	30	2.08%	6.73	**1.01**	98.35%	0.0082	92.20%	0.1679	−5.869E+06	6,024
Flye_1kb	528,563,896	2947	295,613	1,344	93.33%	100	6.94%	31	2.15%	5.70	1.06	94.86%	0.0077	**93.04%**	0.1694	−2.536E+06	7,137
Flye_35kb	516,992,152	2548	385,290	1,336	92.78%	**90**	**6.25%**	31	2.15%	6.50	1.03	94.24%	0.0080	92.34%	0.1699	−2.726E+06	7,458
Marvel_35kb	537,615,613	730	1,202,845	1,180	81.94%	153	10.63%	32	2.22%	3.77	1.08	87.37%	0.0075	85.18%	0.1689	−4.451E+06	5,162
MaSuRCA_1kb	594,528,099	415	3,234,447	**1,362**	**94.58%**	201	13.96%	**21**	**1.46%**	9.27	1.19	94.91%	**0.0060**	91.57%	0.1656	**−1.774E+06**	4,020
MaSuRCA_35kb	594,871,467	416	**3,234,549**	**1,362**	**94.58%**	200	13.89%	**21**	**1.46%**	**9.31**	1.19	94.92%	**0.0060**	91.49%	**0.1655**	−1.790E+06	**4,017**

Note: The best value of each assessment is highlighted in boldface.

Comparison of the 8 metrics we used suggested that the MaSuRCA_35 kb assembly was likely to be the most accurate assembly overall and that the Marvel_35 kb assembly was the least accurate. However, we note that the MaSuRCA assembly did not receive the best scores for all metrics, suggesting that the choice of which assembly to use will sometimes be question-specific. Also, in most cases, performances of the 2 MaSuRCA assemblies are very similar.

N50 scores varied from 295 kb (Flye_1 kb) to 3.2 Mb (MaSuRCA_35 kb), with Flye achieving notably lower N50 values than the other assemblers (Table 3). The low N50 in Flye assemblies is likely to be caused by the high heterozygosity of *E. pauciflora* because Flye is based on using *k*-mers to build an assembly graph, and high heterozygosity will cause differences even among short *k*-mers. BUSCO scores ranged from 1,180 complete genes (81.94%, Marvel_35 kb) to 1,362 complete genes (94.58%, MaSuRCA assemblies), although all assemblies except the Marvel_35 kb assembly had scores >92%. The MaSuRCA_35 kb assembly also achieved the highest LAI score (9.31), which was substantially higher than the best assembly from any other assembler (Canu_1 kb, LAI score: 7.04). The lowest LAI score (3.77) was observed in the Marvel_35 kb assembly. The assembly ploidy was the closest to 1 for the SMARTdenovo assemblies (e.g., 1.01 for the SMARTdenovo_35 kb assembly vs 1.19 for the MaSuRCA_35 kb assembly). These scores have to be interpreted with caution because the true genome size remains unknown; they are to some extent corroborated by the lower number of duplicated BUSCO genes in the assemblies with the lower assembly ploidy (e.g., 100 duplicated BUSCO genes in the SMARTdenovo_35 kb assembly vs 200 in the MaSuRCA_35 assembly). Nevertheless, given that gene duplication is common in *Eucalyptus* species, all such measures need to be interpreted with some caution because the BUSCO genes themselves could be duplicated in the *E. pauciflora* genome. Taken together, these 4 metrics suggest that the MaSuRCA_35 kb assembly is the most complete, the most contiguous, and the most accurate among the assemblies we produced.

The other 3 metrics assess the correctness of every assembly, and they also suggest that the best assemblies for our data were produced by MaSuRCA (Table 3). The MaSuRCA assemblies (1 and 35 kb) had the lowest error rates (0.006 errors per base for short-read mapping and 0.166 for long-read mapping in both assemblies) and the smallest total number of structural variants estimated from the long validation reads (4,017 structural variants for the MaSuRCA_35 kb assembly). Flye and SMARTdenovo assemblies tended to perform the worst on these metrics, although we note that these results will be affected by the fact that the MaSuRCA assemblies contain more duplicated genome regions (see above), which will tend to reduce the estimated error rates and number of structural variants, because duplicated regions can accurately represent heterozygous variants that will be present in the reads. CGAL ranked MaSuRCA assemblies as the best (1 kb likelihood: −1,774,303 and 35 kb likelihood: −1,790,386) and the SMARTdenovo_35 kb assembly as the worst (likelihood: −5,869,476).

Finally, to further investigate the different assemblies, we compared the genome sequence similarity between different assemblies using the NUCmer module of MUMmer (Fig. 4), with the minimum identity set to 75. Notably, ∼8% of the sequence of the Canu/SMARTdenovo/Flye/MaSuRCA assemblies failed to align to the Marvel_35 kb assembly (Fig. 4), which, along with the low genome completeness (BUSCO scores) of the Marvel_35 kb assembly (Table 3), suggests that the Marvel_35 kb assembly may contain many more small duplicated regions than other assemblies. In turn, these duplicated regions may explain the fact that the Marvel_35 kb assembly has the lowest genome completeness but not the smallest genome size compared to other assemblies (Table 3). Other assemblies have ∼97–99% of similarity to each other.

Based on the 8 metrics we used above (Table 3), we suggest that the MaSuRCA_35 kb assembly represents the most accurate representation of the *E. pauciflora* genome. We note, however, that the Flye assembler only took 1–3% of the runtime of the other assemblers used in this article (Table 1) and produced genome assemblies that were of similar quality to the MaSuRCA_35 kb assembly in many respects. The Marvel_35 kb assembly received the worst scores on many metrics and also seems to be missing ∼10% of the genome according to BUSCO scores and genome sequence similarity analyses compared to other assemblies (Table 3).

### Comparative genome analysis between *E. pauciflora* and *E. grandis*

Using the MaSuRCA_35 kb assembly, we estimate that the *E. pauciflora* genome is 594,871,467 bp in length, with 416 contigs and a contig N50 of 3,235 kb. The genome has up to 0.006 errors per base. Approximately 94% of complete BUSCO genes were identified in this *E. pauciflora* genome assembly.

**Figure 5:**
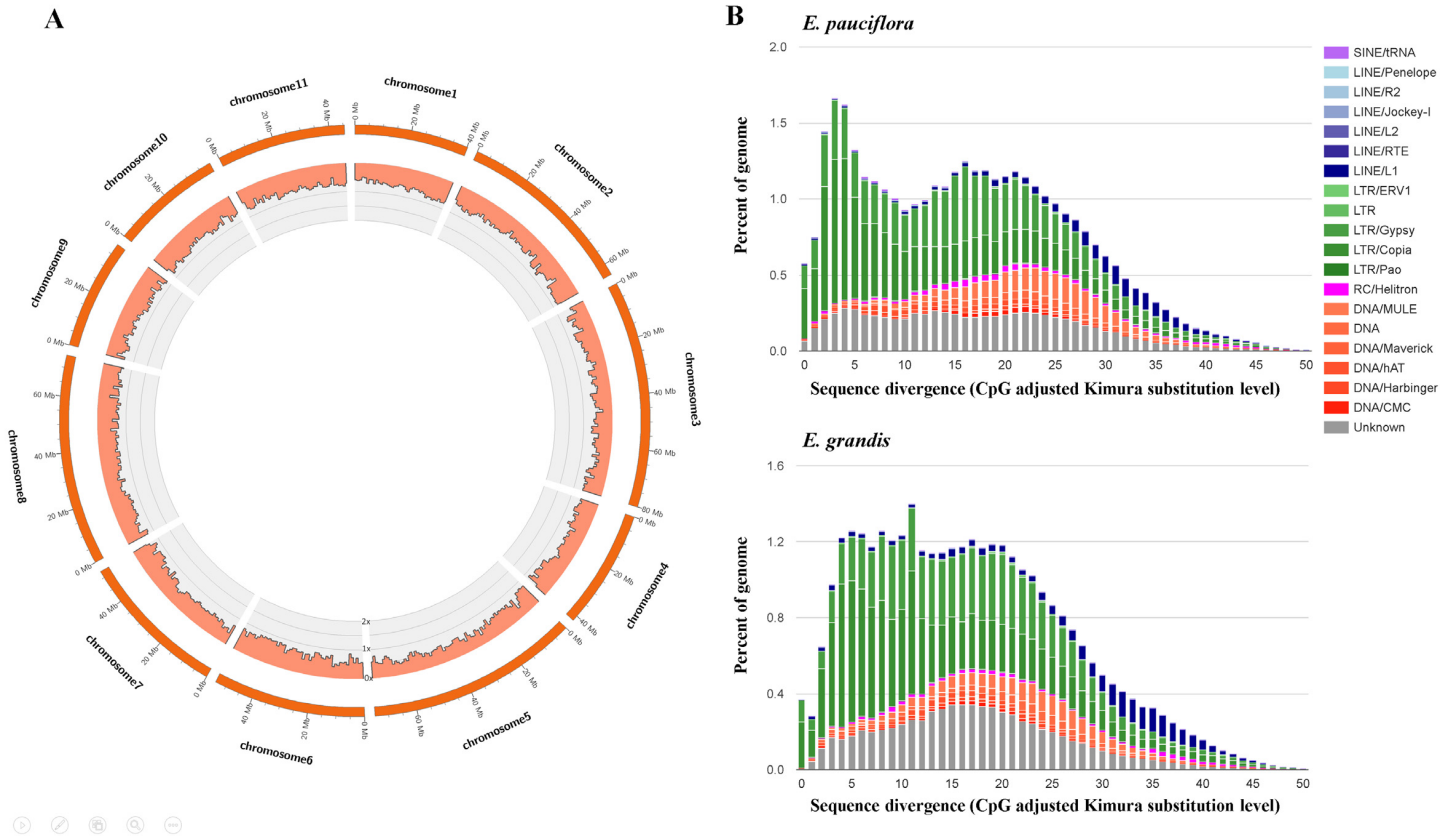
**A**. The histogram of location and coverage of *E. pauciflora* genome aligned to the 11 chromosomes of *E. grandis*. The scale of the y-axis is 0–2× of coverage. Every bar is 1 Mb. The coverage was calculated by the total aligned length of *E. grandis* in each bar/the length of bar. If a site in *E. grandis* is aligned by *E. pauciflora* twice or more, this site will be counted twice or more. **B**. Repeat landscape comparison between *E. pauciflora* and *E. grandis*. Only repeats that are found in both genomes are shown. Older repeat insertions could accumulate more mutations compared to new repeat insertions. This leads older repeat insertions to have accumulated a higher level of divergence (shown on the right side of the graph).

*E. grandis* is the only published *Eucalyptus* genome that is assembled to chromosome level. We therefore compared *E. grandis* with our *E. pauciflora* genome. The *E. grandis* contains 691.43 Mb of sequence, ∼16% larger than the *E. pauciflora* genome. We compared these 2 genome assemblies using the NUCmer module of MUMmer to perform whole-genome alignment as described above. This alignment shows that the *E. pauciflora* genome assembly covers just 61.56% of the *E. grandis* genome sequence, leaving ∼265 Mb of the *E. grandis* genome sequence not covered by the *E. pauciflora* assembly and 113 Mb of the *E. pauciflora* assembly not covered by the *E. grandis* assembly. Despite this, the coverage of the *E. pauciflora* assembly when mapped to the 11 chromosome-scale scaffolds of the *E. grandis* genome is fairly constant (Fig. 5A), suggesting that many of these differences result either from small errors in both assemblies and/or from relatively small-scale differences in the underlying genomes.

To examine whether the differences between *E. pauciflora* and *E. grandis* could be explained by their repeat content, we annotated repetitive elements of *E. pauciflora* and *E. grandis* with RepeatMasker v4.0.7 (RepeatMasker, RRID:SCR_012954) [62]. Although the repeats of *E. grandis* have been annotated before [16], we re-annotated them here to make a direct comparison of the repeat content using an identical pipeline for both genomes. First, we created the custom consensus repeat library using RepeatModeler v1.0.11 (RepeatModeler, RRID:SCR_015027) [63] with parameter “-engine ncbi.” The classifier was built upon Repbase v20170127 [64]. Then we merged the repeat libraries from RepeatModeler and LTR retrotransposon candidates from LTR retriever to create a comprehensive repeat library as the input for RepeatMasker. We ran RepeatMasker with the “-engine ncbi” model. We used the "calcDivergenceFromAlign.pl” script in the RepeatMasker pipeline to calculate the Kimura divergence values, and plotted the repeat landscape with repeats presented in both *E. pauciflora* and *E. grandis* genomes (Fig. 5B).

The repeat content of the 2 genomes is similar. The *E. pauciflora* genome contains 44.77% of repetitive elements, compared to 41.22% in *E. grandis*. Retrotransposons account for 29.53% of the*E. pauciflora* genome, and 26.94% in *E. grandis*, and DNA transposons account for 6.04% and 4.80% of the genome in *E. pauciflora* and *E. grandis*, respectively. Both genomes show ∼2 waves of repeat expansion in the repeat landscapes, which is most likely explained by a shared inheritance of most of the repeats in the 2 genomes (Fig. 5B).

## Conclusions

Here, we report a high-quality draft haploid genome of *E. pauciflora*. It is the first *Eucalyptus* genome assembled with third-generation sequencing reads (Nanopore sequencing) and is the third nuclear genome of *Eucalyptus* species. Due to the economic and ecological importance of *Eucalyptus*, this high-quality genome will support further analysis on *Eucalyptus* and its related species. Finally, the approaches used in this study to assess and compare different assemblies should help in assessing and choosing among many potential genome assemblies.

## Availability of Supporting Data and Materials

The *E. pauciflora* genome project was deposited at NCBI under BioProject number PRJNA450887. The whole-genome sequencing data are available in the SRA with accession number SRR7153044-SRR7153116. The scripts we used in this article, including the genome assembly, genome polishing, repeat annotation, and genome assessments, are available on Github [65]. Also, a single universal pipeline containing the assessment methods we used in this article is available on Github [66]. All supporting data and materials are available in the *GigaScience* GigaDB database [67].

## Additional Files

**Fig. S1**. GenomeScope result of *E. pauciflora*

**Fig. S2**. Genome contamination detection. Almost all sequences were matched to the sequences in the streptophyta phylum group. No contamination was found.

**Supplementary Results**

**Table S1**. The comparison of assemblies with corrected and uncorrected long-read datasets

**Table S2**. The comparison of polishing results of raw assemblies

**Table S3**. The comparison of polishing results of each genome after haplotig removal

giz160_GIGA-D-19-00372_Original_Submission

giz160_GIGA-D-19-00372_Revision_1

giz160_Response_to_Reviewer_Comments_Original_Submission

giz160_Reviewer_1_Report_Original_Submission Jue Ruan -- 11/13/2019 Reviewed

giz160_Reviewer_2_Report_Original_Submission Alexey Gurevich -- 11/15/2019 Reviewed

giz160_Supplemental_Figures_and_Tables

## Abbreviations

bp: base pairs; BUSCO: Benchmarking Universal Single-Copy Orthologs; CGAL: computing genome assembly likelihoods; CPU: central processing unit; CTAB: cetyl trimethylammonium bromide; Gb: gigabase pairs; GCICA: gene conservation informed contig alignment; kb: kilobase pairs; LAI: long-terminal repeat assembly index; LTR: long-terminal repeat; MaSuRCA: Maryland Super Read Cabog Assembler; Mb: megabase pairs; NCBI: National Center for Biotechnology Information; RAM: random access memory. REAPR: Recognition of Errors in Assemblies using Paired Reads; SRA: Sequence Read Archive.

## Competing Interests

The authors declare that they have no competing interests.

## Ethics Statement

*E. pauciflora* leaves were collected from a single *E. pauciflora* tree in Thredbo, Kosciuszko National Park, New South Wales, Australia (Latitude − 36.49433, Longitude 148.282983). The written permission was from the Scientific Licensing office of the Office of Environment and Heritage for New South Wales: www.licence.nsw.gov.au, in accordance with national guidelines in Australia. Tissues were not deposited as voucher specimens.

## Funding

This research is supported by the Australian Research Council Future Fellowship, FT140100843 to R.L. and FT180100024 to B.S..

## Authors' Contributions

A.D., D.K., R.L., and W.W. conceived this project. A.M.S. and R.L. performed sample collection for Illumina sequencing. A.M.S. extracted genomic DNA and constructed libraries for Illumina sequencing. R.L. and M.S. carried out sample collection for Nanopore sequencing. M.S. and B.S. performed DNA extraction, library preparation, and Nanopore sequencing. D.K. performed long-read polishing and Canu 1 kb assembly, whereas A.D. performed Canu_35 kb, Flye_1 kb, Flye_35 kb, and Marvel_35 kb assemblies and contamination detection. A.D. and W.W. conducted the whole-genome alignment analysis. W.W. conducted all the remaining analyses. A.D., B.S., D.K., R.L., and W.W. were involved in data interpretation. A.D., R.L., and W.W. drafted the original manuscript. R.L. and W.W. finalized the manuscript. All authors read and approved the final manuscript.
