# Supplementary material for: The draft nuclear genome assembly of Eucalyptus pauciflora: a pipeline for comparing de novo assemblies
Source: Gigascience. 2020 Jan 2;9(1):giz160. doi: 10.1093/gigascience/giz160 (PMC6939829; doi:10.1093/gigascience/giz160)
Supplement: giz160_GIGA-D-19-00372_Original_Submission [file giz160_giga-d-19-00372_original_submission.pdf]

GigaScience

The draft nuclear genome assembly of Eucalyptus pauciflora: a pipeline for comparing de novo assemblies

--Manuscript Draft--

Manuscript Number:	GIGA-D-19-00372
Full Title:	The draft nuclear genome assembly of Eucalyptus pauciflora: a pipeline for comparing de novo assemblies
Article Type:	Data Note
Funding Information:	Australian Research Council Future Fellowship (FT140100843) — Dr Robert Lanfear<br>Australian Research Council Future Fellowship (FT180100024) — Dr Benjamin Schwessinger
Abstract:	Background<br>Eucalyptus pauciflora (the snow gum) is a long-lived tree with high economic and ecological importance. Currently, little genomic information for Eucalyptus pauciflora is available. Here, we sequentially assemble the genome of Eucalyptus pauciflora with different methods, and combine multiple existing and novel approaches to help to select the best genome assembly.<br>Findings<br>We generated high coverage of long- (Nanopore, 174x) and short- (Illumina, 228x) read data from a single Eucalyptus pauciflora individual and compared assemblies from five assemblers (Canu, SMARTdenovo, Flye, Marvel, and MaSuRCA) with different read lengths (1kb and 35 kb minimum read length). A key component of our approach is to keep a randomly selected collection of ~10% of both long- and short-reads separated from the assemblies to use as a validation set for assessing assemblies. Using this validation set along with a range of existing tools, we compared the assemblies in eight ways: contig N50, BUSCO scores, LAI (Long terminal repeat Assembly Index) scores, assembly ploidy, base-level error rate, CGAL (Computing Genome Assembly Likelihoods) scores, structural variation, and genome sequence similarity. Our result showed that MaSuRCA generated the best assembly, which is 594.87 Mb in size, with a contig N50 of 3.23 Mb, and an estimated error rate of ~0.006 errors per base.<br>Conclusions<br>We report a draft genome of Eucalyptus pauciflora , which will be a valuable resource for further genomic studies of eucalypts. The approaches for assessing and comparing genomes, should help in assessing and choosing among many potential genome assemblies from a single dataset.
Corresponding Author:	Weiwen Wang<br>Australian National University Research School of Biology<br>Canberra, ACT AUSTRALIA
Corresponding Author Secondary Information:	
Corresponding Author's Institution:	Australian National University Research School of Biology
Corresponding Author's Secondary Institution:	
First Author:	Weiwen Wang
First Author Secondary Information:	
Order of Authors:	Weiwen Wang<br>Ashutosh Das<br>David Kainer<br>Miriam Schalamun

Powered by Editorial Manager® and ProduXion Manager® from Aries Systems Corporation

	Alejandro Morales-Suarez
	Benjamin Schwessinger
	Robert Lanfear
Order of Authors Secondary Information:	
Additional Information:	
**Question**	**Response**
Are you submitting this manuscript to a special series or article collection?	No
**Experimental design and statistics**<br><br>Full details of the experimental design and statistical methods used should be given in the Methods section, as detailed in our Minimum Standards Reporting Checklist. Information essential to interpreting the data presented should be made available in the figure legends.<br><br>Have you included all the information requested in your manuscript?	Yes
**Resources**<br><br>A description of all resources used, including antibodies, cell lines, animals and software tools, with enough information to allow them to be uniquely identified, should be included in the Methods section. Authors are strongly encouraged to cite Research Resource Identifiers (RRIDs) for antibodies, model organisms and tools, where possible.<br><br>Have you included the information requested as detailed in our Minimum Standards Reporting Checklist?	Yes
**Availability of data and materials**<br><br>All datasets and code on which the conclusions of the paper rely must be either included in your submission or deposited in publicly available repositories (where available and ethically	Yes

appropriate), referencing such data using a unique identifier in the references and in the "Availability of Data and Materials" section of your manuscript.<br><br>Have you have met the above requirement as detailed in our Minimum Standards Reporting Checklist?	

Powered by Editorial Manager® and ProduXion Manager® from Aries Systems Corporation

Manuscript Click here to download Manuscript Epau_genome_v2.docx

The draft nuclear genome assembly of *Eucalyptus pauciflora*: a pipeline for comparing *de novo* assemblies

Weiwen Wang[1*^], Ashutosh Das[1,2^], David Kainer[1], Miriam Schalamun[1,3], Alejandro Morales-Suarez[4], Benjamin Schwessinger[1], Robert Lanfear[1*]

1. Research School of Biology, the Australian National University, Canberra, Australia
2. Department of Genetics and Animal Breeding, Faculty of Veterinary Medicine, Chittagong Veterinary and Animal Sciences University, Chittagong, Bangladesh
3. Institute of Applied Genetics and Cell Biology, University of Natural Resources and Life Sciences, Vienna, Austria
4. Department of Biological Sciences, Macquarie University, Sydney, Australia

^ Equal contribution

* Corresponding authors: wei.wang@anu.edu.au and rob.lanfear@anu.edu.au

Email:

Weiwen Wang: wei.wang@anu.edu.au

Ashutosh Das: ashutosh.das@cvasu.ac.bd

David Kainer: dkainer@outloolk.com

Miriam Schalamun: miriam.schalamun@gmail.com

Alejandro Morales-Suarez: eder-alejandro.morales-suar@hdr.mq.edu.au

Benjamin Schwessinger: benjamin.schwessinger@anu.edu.au

Robert Lanfear: rob.lanfear@anu.edu.au

Abstract

Background

Eucalyptus pauciflora (the snow gum) is a long-lived tree with high economic and ecological importance. Currently, little genomic information for *Eucalyptus pauciflora* is available. Here, we sequentially assemble the genome of *Eucalyptus pauciflora* with different methods, and combine multiple existing and novel approaches to help to select the best genome assembly.

Findings

We generated high coverage of long- (Nanopore, 174x) and short- (Illumina, 228x) read data from a single *Eucalyptus pauciflora* individual and compared assemblies from five assemblers (Canu, SMARTdenovo, Flye, Marvel, and MaSuRCA) with different read lengths (1kb and 35 kb minimum read length). A key component of our approach is to keep a randomly selected collection of ~10% of both long- and short-reads separated from the assemblies to use as a validation set for assessing assemblies. Using this validation set along with a range of existing tools, we compared the assemblies in eight ways: contig N50, BUSCO scores, LAI (Long terminal repeat Assembly Index) scores, assembly ploidy, base-level error rate, CGAL (Computing Genome Assembly Likelihoods) scores, structural variation, and genome sequence similarity. Our result

showed that MaSuRCA generated the best assembly, which is 594.87 Mb in size, with a contig N50 of 3.23 Mb, and an estimated error rate of ~0.006 errors per base.

Conclusions

We report a draft genome of *Eucalyptus pauciflora*, which will be a valuable resource for further genomic studies of eucalypts. The approaches for assessing and comparing genomes, should help in assessing and choosing among many potential genome assemblies from a single dataset.

Keywords: Long-read assembly; nanopore sequencing; hybrid assembly; genome assessment; assembly comparison; *Eucalyptus pauciflora*; haplotig separation; genome polishing

Data Description

Introduction

Eucalypts are widely distributed in Australia, including three genera *Eucalyptus*, *Corymbia* and *Angophora*, and have around 900 species [1]. *Eucalyptus pauciflora* (*E. pauciflora*) (Fig. 1), also known as snow gum, is a highly variable eucalyptus species that inhabits diverse landscapes in south-eastern Australia [1]. *E. pauciflora* can survive from close to sea level to up to the tree line of the Australian Alps, displaying the broadest altitudinal range in the *Eucalyptus* genera [2-4]. Due to its wide distribution and drought and cold tolerance, *E. pauciflora* is used for carbon offset plantings,

ecological restoration, honeybee food source, and also has medicinal uses [1, 5-11]. However, genomic resources for *E. pauciflora* are currently very limited: there exists a single chloroplast genome [12], two sets of microsatellite markers [13, 14], and two nuclear loci used for phylogenetics [15]. The assembly of *E. pauciflora* genome will assist in elucidating the genetic basis of drought and cold tolerance in *Eucalyptus*.

Across the ~900 extant eucalypt species, there are only two genomes published: those for *E. camaldulensis* and *E. grandis* [16, 17]. Both of these genomes were sequenced with a combination of Sanger sequencing and short-read sequencing, and as a result both assemblies are somewhat fragmented. There are 81,246 scaffolds in *E. camaldulensis* assembly [17]. While the *E. grandis* genome is highly contigous, assembled to chromosome level, it still has 4,941 unplaced scaffolds [16]. New technologies, such as third-generation long-read sequencing, have the potential to produce less fragmented assemblies at a fraction of the cost of previous methods. Nevertheless, many challenges still remain, not least of which is that different genome assembly software, and small changes to the parameters of a single piece of software, can produce substantially different assemblies. In light of this, methods for choosing the most accurate assembly from a set of possible assemblies have become increasingly important.

Two metrics are commonly used to assess and compare genome assemblies: contig N50 and Benchmarking Universal Single-Copy Orthologs (BUSCO [18],

RRID:SCR_015008) scores. The contig N50 is the size of the contig such that at least 50% of the assembled nucleotides can be found in contigs of that size or larger. The N50 is a measure of genome contiguity, where a higher N50 suggests a genome that has been assembled into fewer and larger contigs. All else being equal, we should prefer genome assemblies with a larger N50, up to the point where the N50 is equal to the N50 of the chromosomes themselves. Perhaps because of this, the N50 is one of the most widely reported metrics in genome assembly. However, it is important to remember that the N50 measures contiguity, not accuracy. For example, N50 scores may be artificially inflated by incorrectly linking contigs [19, 20]. The BUSCO score estimates the proportion of highly conserved orthologous genes that are present in assemblies. The underlying assumption is that there exists a certain set of highly conserved single-copy genes, the vast majority of which we should expect to observe in single copies in any given haploid genome assembly. BUSCO scores provide a very useful measure of genome assembly completeness (a component of accuracy), and in principle we should prefer genome assemblies with BUSCO scores closer to 100%. One limitation of BUSCO scores is that they assess only a very small proportion of the genome, typically around 1000 highly conserved genes which represent less than 1% of the total genome. Furthermore, by their nature these protein-coding regions of the genome tend to be among the easiest to assemble because they are usually single-copy regions. Hence, assemblies can have very similar BUSCO scores even if they differ considerably in their assembly of the non-BUSCO genomic regions, which means that it is sometimes difficult to use BUSCO scores to distinguish among competing assemblies [21]. In this

study, we complement these commonly-used measures with a range of other metrics to assess and compare genome assemblies, and we use these measures to choose the best draft assembly of *E. pauciflora*.

One measure we propose is the assembly ploidy: the proportion of the genome that is represented by haploid contigs. One important problem in genome assembly is that we commonly represent the genome of diploid (or polyploid) organisms as a haploid sequence. Traditionally, genome projects would alleviate this problem by sequencing highly inbred individuals [22, 23], thus reducing the discrepancy between the diploid individual and the haploid representation. However, as genome assembly has become more commonplace, we often want to assemble the genomes of highly heterozygous individuals. For example, heterozygosity in *Eucalyptus* is around 1% [24], and varies substantially along the genome [16]. The consequence of this is that regions of low heterozygosity tend to be assembled into a single collapsed haploid sequence, whereas regions of high heterozygosity tend to be assembled into two haplotypes of the same region, which are usually labelled the 'primary contig' (referring to the longer of the two contigs) and the 'haplotig' (referring to the shorter of the two contigs) [25]. Although there has been some progresses in estimating truly diploid assemblies [25, 26], most assemblers still produce primary contigs and haplotigs without labelling them as such [27, 28]. Crucially, unidentified haplotigs may cause issues in the downstream analyses, because many analyses assume that we have a haploid representation of the genome. Because of this, we propose a novel and simple (but imperfect) metric to

measure the assembly ploidy, which is simply the ratio of the assembly size to the estimated haploid genome size. If the aim is to produce a haploid representation of a genome, then an assembly ploidy of 1 is preferable (i.e. the assembly size should be equal to the estimated haploid genome size). If the aim is to produce a diploid representation of a genome, then an assembly ploidy of 2 is preferable (i.e. the assembly size should be double the estimated haploid genome size). One limitation of this metric is that it is sensitive to errors in the estimation of haploid genome size, and it is also sensitive to errors in genome assembly (e.g. highly incomplete assemblies) that might affect the numerator. Nevertheless, in combination with other measures, we show below that the assembly ploidy provides a useful metric for comparing genome assemblies.

We also apply a suite of measures designed to provide a genome-wide assessment of contiguity and accuracy that can complement the widely-used contig N50 and BUSCO scores. The advantages of these measures lie in the fact that they assess more of the genome than BUSCO scores, though each also has its limitations. Serval tools have been developed to evaluate the quality of assemblies given an alignment of sequencing reads to the assembly, including FRCbam (FRCbam, RRID:SCR_005189) [29], Recognition of Errors in Assemblies using Paired Reads (REAPR) [30], and Computing Genome Assembly Likelihoods (CGAL) [20]. All of these tools require read alignment information. FRCbam first computes a series of features with the alignment information, and then creates feature response curves that can be used to assess and compare assemblies. REAPR uses the read alignment to identify possibly

misassembled regions and to give a score for the accuracy of each base in the genome. CGAL provides the likelihood of an assembly, calculated from a model that accounts for errors in reads, read coverage across the assembly, and the proportion of reads that do not contribute to the assembly. Of these three related tools, we use CGAL in this study because it provides a single likelihood score for each assembly, such that a higher likelihood from CGAL suggests that a genome assembly is a better representation of the truth, making it very simple to compare multiple assemblies. The second measure we used is the long-terminal repeat (LTR) assembly index, or LAI [21]. The LAI score is the proportion of LTR sequences in the genome that are intact, and is independent of genome size and repeat content. In general, a higher LAI score suggests a more contiguous and complete assembly [21]. The third measure we use is the base-level error rate evaluated by remapping independent sets of long and short validation reads (around 10% of all reads, randomly selected) to the assembly. Previous studies have evaluated the base-level error rate by remapping all reads to the assembly [31, 32]. Here, we use validation reads which are not involved in the assembly, in order to avoid any possible biases introduced by validating an assembly with the same data that was used to produce it. For a perfect assembly in which the ploidy of the entire assembly matches the ploidy of the individual, a lower base-level error rate is preferable, with a theoretical minimum of the error rate of the sequencing technology (e.g. ~0.3% for raw Illumina reads [33], and ~10-15% for raw Nanopore reads [34, 35]). For a haploid representation of a diploid assembly, the minimum possible base-level error rate will be higher, because by necessity a haploid representation of a heterozygous site will not match

approximately half of the reads. In this case, the theoretical minimum base-level error rate is the sum of the error rate of the sequencing technology and half of the heterozygosity. The fourth measure we use is the number of structural variants detected when re-mapping our long validation reads to assemblies. As with the base-level error rate, if the ploidy of the assembly matches the ploidy of the individual, then the theoretical minimum of this metric is the structural error rate introduced into sequencing reads by the sequencing technology. For a haploid representation of a diploid genome, the theoretical minimum is the sum of the error rate of the technology plus half of the structural heterozygosity. These two quantities are rarely known, but nevertheless, a very high structural error rate of validation reads mapped to a haploid assembly may indicate cases in which the assembly has a large proportion of incorrectly linked contigs. The final measure is the genome sequence similarity of each assembly when compared to all other assemblies. This measure does not provide any information relative to an underlying truth, but it may help to identify significant differences between otherwise plausible genome assemblies that can aid in choosing the best assembly. The selection of the best assembly should consider all measures together.

Here, we used long- and short-reads to create a draft haploid assembly of the *E. pauciflora* genome. We use the metrics we describe above to compare a range of assemblies from a range of different assemblers. We performed different assemblies with long-read-only assemblers (Canu (Canu, RRID:SCR_015880) [36], SMARTdenovo [37], Flye (Flye, RRID:SCR_017016) [38] and Marvel [39]) and a

hybrid assembler MaSuRCA (MaSuRCA, RRID:SCR_010691) [40], using long-read datasets with different minimum read lengths in each case (1 kb and 35 kb).

Sample collection, DNA sequencing and quality control

We collected leaves from the single *E. pauciflora* tree near Thredbo, Kosciuszko National Park, New South Wales, Australia (36° 29′ 39.58″ N, 148° 16′ 58.73″ E) in March 2016 (for Illumina sequencing) and June 2017 (for MinION sequencing). We stored leaves at 4°C when transported them to the laboratory.

For long-read sequencing, we extracted high molecular weight genomic DNA from leaves following a protocol optimized for *Eucalyptus* nanopore sequencing [41]. We prepared ONT 1D ligation libraries according to the manufacturer's protocol (SQK-LSK108) and sequenced the reads using MinKNOW v1.7.3 with R9.5 flowcells on a MinION sequencer. We performed basecalling with Albacore v2.0.2 (Albacore, RRID:SCR_015897). This resulted in 12,584,100 raw long-reads (106.96 Gb) with average read length of 8.5 kb. We removed adapters from long-reads with Porechop v0.2.1 (Porechop, RRID: SCR_016967) [42]. Next, we trimmed bases with quality <10 on both ends of the reads using NanoFilt v2.0.0 (NanoFilt, RRID:SCR_016966) [43] and discarded reads shorter than 1 kb after trimming. This recovered 96.66 Gb of long-read data comprising 7,711,141 filtered reads with an average read length of 12.53 kb (minimum 1 kb and maximum ~150 kb). Given an estimated genome size of 500 Mb

(see below), this represents a coverage of 193x.

For short-read sequencing, we extracted genomic DNA from freeze-dried leaves using a CTAB protocol [44] followed by purification with a Zymo kit (Zymo Research Corp). We constructed TruSeq Nano libraries with an insert size of 400 bp using protocol provided by Illumina, then sequenced the reads (paired-end 150 bp) using an Illumina Hiseq2500 platform (Illumina Inc., San Diego, CA). This Illumina sequencing generated 506,840,789 paired raw reads (152.05 Gb). We used BBDuk v37.31 (BBmap, RRID:SCR_016965) [45] to remove adapters and to trim both sides of raw short-reads which quality was lower than 30. We discarded filtered reads with a length under 50 bp. Around 122.69 Gb short-read data containing 414,697,585 paired reads were left, representing 246x coverage with an estimated genome size of 500 Mb (see below).

Genome size estimation

We used GenomeScope (GenomeScope, RRID:SCR_017014) [46] and SGA-preqc (SGA, RRID:SCR_001982) [47] to estimate the *E. pauciflora* genome size. We first generated a 32-mer distribution using Jellyfish v1.1.12 (Jellyfish, RRID:SCR_005491) [48] from all of our short-reads, then ran GenomeScope using this 32-mer distribution with a maximum k-mer coverage of 1000x. This gave a genome size estimate of 408.16 Mb (Additional file 1: Fig. S1), which is lower than expected for other *Eucalyptus* species [16, 17]. However, it is known that genomic repeats can lead to underestimation of genome sizes from uncorrected kmer distributions [49], and the *Eucalyptus* genome

is repeat-rich, for example around 50% of genome was annotated as repeats in *E. grandis* [16], suggesting that 408.16 Mb may be a significant underestimate of the genome size. Also, GenomeScope suggests that the heterozygosity of *E. pauciflora* is 1.5%. SGA-preqc estimates genome size from k-mer distributions that are corrected to attempt to better account for repeat content, in line with this, SGA-preqc gave a genome size estimate of 529.40 Mb. Because of this, we expect that the SGA-preqc genome size is likely to be more accurate, and in what follows we assume that the *E. pauciflora* genome size is roughly 500 Mb. This suggests that the *E. pauciflora* genome may be around ~30% smaller than that of the other two sequenced *Eucalyptus* species, *E. grandis* (691.43 Mb) [16] and *E. camaldulensis* (654.92 Mb) [17]. However, the genome sizes of *E. grandis* and *E. camaldulensis* may be overestimated due to the assembly and scaffolding of both haplotypes at high heterozygous regions.

Creation of assembly and validation datasets

We separated our long-read and short-read data into assembly and validation datasets by randomly assigning the trimmed and filtered reads into the two datasets with custom scripts [50]. The assembly dataset comprised 86.98 Gb of long-read data (174x coverage) and 114.10 Gb of short-read data (228x coverage). The validation dataset comprised and 9.67 Gb of long-read data (19x coverage, 10% of total long-reads) and 8.59 Gb of short-read data (17x coverage, 7% of total short-reads).

Genome assembly

Here, we compared seven long-read-only assemblies and two hybrid assemblies. For each combination of data and genome assembler, we followed the same genome assembly pipeline. We first used the assembler to produce an initial assembly. Following this, we identified and removed contigs from contaminant sequences, and then polished the resulting assembly. We then identified and removed haplotigs from the assembly. Each assembly was re-polished after haplotig removal. To select the best assembly, we calculated the contig N50 with Quast v4.6.0 (QUAST, RRID:SCR_001228) [19], BUSCO scores with BUSCO v3.0.2, and LAI scores using the LTR_retriever pipeline [51]. After mapping the long- and short- validation reads to the final assemblies (using Ngmlr v0.2.6 [52] for the former and Bowtie2 v2.3.4.1 (Bowtie2, RRID:SCR_016368) [53] for the latter), we calculated the base-level error rate using QualiMap v2.2.1 (QualiMap, RRID:SCR_001209) [54], the structural variant error rate using Sniffles v1.0.8[52], and CGAL scores using CGAL. Finally, we performed whole genome alignment between different assemblies with NUCmer module of MUMmer v4.0.0beta2 [55].

Oxford Nanopore reads tend to have error rates of ~10-15%, which can make assembly of uncorrected reads very challenging. To alleviate this, we first corrected the long-reads assembly dataset with Canu v1.6 with default parameters except for setting corMinCoverage to 8, meaning that read correction would only be applied where at least 8 reads overlapped. We deemed this reasonable given the very high coverage of our data (174x). We then put the corrected long-read datasets into two sets for assembly.

The first dataset contained all corrected long-reads, such that the minimum read length was 1 kb (174x of coverage). The second dataset contained all corrected reads longer than 35 kb (~40x of coverage). We refer to these datasets as the 1 kb and the 35 kb datasets, respectively.

We first compared the performance of using corrected and uncorrected long-reads and uncorrected long-reads to assemble the genome with two efficient assemblers, Flye v2.3.5 and wtdbg2 v2.5 (WTDBG, RRID:SCR_017225) [56] (Additional file 3: Supplementary result). The results showed clearly that corrected long-reads produced better assemblies than uncorrected long-reads using Flye, while the differences with wtdbg2 were less pronounced (Table S1). Nevertheless, the Flye assemblies with corrected reads were the best overall, so we therefore decided to use corrected long-reads for the rest of the assemblies in the study.

We attempted eight long-read-only assemblies and two hybrid assemblies. Assemblies solely with long-read data were performed on corrected reads of two read lengths (1 kb and 35 kb) using four long-read assemblers: Canu v1.6 and v1.7, SMARTdenovo, Flye v2.3.5 and Marvel v1.0. The Marvel assembly with 1kb dataset was not feasible because it required more disk space than we had available, resulting in seven successful long-read only assemblies. We used MaSuRCA v3.2.6 to perform hybrid assemblies with both read length datasets (1 kb and 35 kb) each combined with the short-read dataset. In what follows, we refer to these assemblies as Canu_1kb, Canu_35kb,

SMARTdenovo_1kb, SMARTdenovo_35kb, Flye_1kb, Flye_35kb, Marvel_35kb, MaSuRCA_1kb and MaSuRCA_35kb. In general, we used default settings in all assemblers, and an estimated genome size of 500 Mb where this setting was required. For Canu assemblies, the 1 kb dataset was assembled using Canu v1.6, whereas the 35 kb dataset was assembled using Canu v1.7. We did not repeat the Canu_1kb assembly after Canu v1.7 was released, because we no longer had sufficient computational resources. For Flye assembler, we used the "nano-cor" parameter which accounts for the use of corrected nanopore reads. The chloroplast genome and mitochondrial genome were removed from each assembly by searching for the relevant contigs using BLASTN v2.7.1+ (BLASTN, RRID:SCR_001598) [57] with an E-value cutoff of at most $1x10^{-20}$. For each assembly, we recorded the runtime in CPU hours, the raw assembly length, and the N50 (Table 1).

Contamination detection

Following initial assembly, we used Blobtools v1.0.1 [58] to assess contamination in each genome assembly. To do this, we first generated a hit file for each assembly by searching all contigs against the National Center for Biotechnology Information (NCBI) non-redundant nucleotide database using BLASTN v2.7.1+ (E-value $\leq 1x10^{-20}$). We then analysed the hit file for each assembly using Blobtools, which provides taxonomic annotations and other diagnostic plots to detect contamination in raw genome assemblies. The top-hit was streptophyta phylum, comprising 99.72% to 100% of the hits in different assemblies (Additional file 2: Fig. S2), indicating that there was no

potential contamination from a non-plant origin in each raw assembly.

Genome polishing

We polished each initial genome assembly in order to improve its accuracy. For the Canu, SMARTdenovo, Flye, and Marvel assemblies (i.e. those built from long-reads only), we polished first with Racon v0.5 [59] using Ngmlr using the long-read assembly dataset, and then with Pilon v1.22 (Pilon, RRID:SCR_014731) [60] using Bowtie2 with the short-read assembly dataset. For the MaSuRCA assemblies, we polished only with Pilon because MaSuRCA is a hybrid assembler, and using error-prone long-reads to polish hybrid assemblies tends to induce more errors rather than remove them (Additional file 5: Table S2).

We ran each polishing algorithm for multiple iterations until the accuracy of the resulting assembly stopped improving or improving slightly. We assessed the improvements using BUSCO scores and the base-level error rate by re-mapping validation long- and short-reads to each assembly (mapped as above). We evaluated the BUSCO scores using BUSCO with the embryophyta_odb9 lineage (1440 genes in total). Polishing with Racon took between 2 and 12 iterations, and with Pilon between 3 and 10 iterations (Additional file 5: Table S2).

Polishing with both Racon and Pilon significantly improved all of the raw genome assemblies, measured with base-level errors in long- and short- reads, and with BUSCO

scores (Additional file 5: Table S2). Polishing with Racon improved long-read base level accuracy by up to 0.83% (in the Marvel_35kb assembly), short-read base level accuracy by up to 1.51% (also in the Marvel_35kb assembly), and the BUSCO completeness scores by up to 30.76% (in the Flye_35kb assembly). Polishing with Pilon further improved the long-read base level accuracy by up to 0.40% (in the Marvel_35kb assembly), the short-read base level accuracy by up to 1.41% (in the Flye_35kb assembly), and the BUSCO completeness scores by up to 24.44% (in the Flye_1kb assembly).

Assembly ploidy and haplotig removal

Comparison of the polished genome assemblies revealed large variation in assembly size (Table 2). We calculated the assembly ploidy of each assembly as described above, assuming a genome size of 500 Mb. The assembly ploidy ranges from 1.12 (Flye_35kb assembly) to 1.79 (Canu_1kb assembly) (Table 2), suggesting that the Canu_1kb assembly is close to a diploid assembly (i.e. ~80% of the genome is represented by two contigs) and that the Flye_35kb assembly is close to a haploid assembly (i.e. only ~12% of the genome is represented by two contigs). To attempt to produce haploid representations of the genome from all assemblies, we used Purge Haplotigs [28] and a custom pipeline, which we call gene conservation informed contig alignment (GCICA) (script available on Github from [61]), to find and remove haplotigs from all the assemblies (Fig. 2A).

Purge Haplotigs assigns contigs to primary contigs and haplotigs depending on both coverage information generated by long-read mapping and pairwise alignments of all contigs. To run Purge Haplotigs, we first mapped the long-read assembly dataset to each polished assembly using Ngmlr, and then separated the contigs into primary contigs and haplotigs with default settings. 8% to 29% of each genome assembly (after polishing) was annotated as haplotigs, and removing these haplotigs reduced the assembly ploidy from 1.12 – 1.79 to 1.01 – 1.24 (Table 2).

The high assembly ploidy for some assemblies after running Purge Haplotigs suggested that these assemblies retained haplotigs that covered up to 29% of the genome. We therefore further filtered possible haplotigs using a custom approach, GCICA. If a pair of contigs comprise a primary contig and a haplotig, we would expect most of regions of the haplotig to be very similar to that of the primary contig. To find putative pairs of primary contigs and haplotigs, we therefore looked for pairs of contigs with similar gene content, and then examined these pairs in more detail. To do this, we first mapped the nucleotide sequences of all *E. grandis* genes to all contigs in an assembly using BLASTN (E-value ≤ $1x10^{-5}$). If >70% of mapped markers in a contig could also be mapped to another contig, and at least 80% of sequence of the smaller contig could be aligned to the other contig (detecting with NUCmer module of MUMmer), we considered these two contigs as a putative primary contig and haplotig pair. We then examined the alignments of all such pairs by eye and removed any pairs in which the smaller contig appeared to be completely contained within the larger, i.e. in which the

smaller contig was an unambiguous haplotig. This process identified a further ~0-2% of each assembly as haplotigs (Table 2).

Following removal of haplotigs, we re-evaluated each assembly using BUSCO scores (Fig. 2B and 2C). We noted that, depending on the genome assembly, the number of complete BUSCO genes sometimes dropped and sometimes increased slightly after removing haplotigs (Fig. 2B). We hypothesised that BUSCO scores could drop either because haplotig removal mistakenly removed a contig that was not a haplotig, or because haplotig removal correctly removed a haplotig which contained a more conserved representation of a BUSCO gene. BUSCO scores could increase because they are based on E-value scores of alignments, which may be affected by the total length of the assembly. To attempt to alleviate some of these potential issues, we re-polished all of the genome assemblies with multiple rounds of Pilon using the short-read assembly dataset, as above. BUSCO scores recovered across all assemblies with additional Pilon polishing (Fig. 2B). As expected, the number of duplicated BUSCO genes decreased substantially (~50%-70%) after haplotigs were removed from the assemblies and this did not change substantially after additional polishing (Fig. 2C and Additional file 6: Table S3). Together, these results suggest that our haplotig removal pipelines largely succeeded in removing haplotigs, although some haplotigs likely remain if the true genome size is around 500 Mb (Fig. 2A).

Assessment of assembly quality with eight measures

After haplotig removal and polishing, we considered the primary contigs of each assembly as the final assembly, and evaluated each of the final assembly in using the eight statistics we describe above: contig N50, BUSCO scores, LAI scores, assembly ploidy, base-level error rate, CGAL scores, structural variation and genome sequence similarity (Table 3 and Figs. 3 and 4).

Comparison of the eight metrics we used suggested that the MaSuRCA_35kb assembly was likely to be the most accurate assembly overall and that the Marvel_35kb assembly was the least accurate. However, we note that the MaSuRCA assembly did not receive the best scores for all metrics, suggesting that the choice of which assembly to use will sometimes be question-specific. Also, in most of cases, performances of the two MaSuRCA assemblies are very similar.

N50 scores varied from 295 kb (Flye_1kb) to 3.2 Mb (MaSuRCA_35kb), with Flye achieving notably lower N50 values than the other assemblers (Table 3). The low N50 in Flye assemblies is likely to be caused by the high heterozygosity of *E. pauciflora*, because Flye is based on using k-mer to build an assembly graph, and high heterozygosity will cause differences even among short k-mers. BUSCO scores ranged from 1180 complete genes (81.94%, Marvel_35kb) to 1362 complete genes (94.58%, MaSuRCA assemblies), although all assemblies except the Marvel_35kb assembly had scores >92%. The MaSuRCA_35kb assembly also achieved the highest LAI score (9.31), which was substantially higher than the best assembly from any other assembler

(Canu_1kb, LAI score: 7.04). The lowest LAI score (3.77) was observed in Marvel_35kb assembly. The assembly ploidy was the closest to one for the SMARTdenovo assemblies (e.g.1.01 for the SMARTdenovo_35kb assembly vs. 1.19 for the MaSuRCA_35kb assembly). These scores have to be interpreted with caution, because the true genome size remains unknown, they are to some extent corroborated by the lower number of duplicated BUSCO genes in the assemblies with the lower assembly ploidy (e.g. 100 duplicated BUSCO genes in the SMARTdenovo_35kb assembly vs. 200 in the MaSuRCA_35 assembly). Nevertheless, given that gene duplication is common in *Eucalyptus* species, all such measures need to be interpreted with some caution, since the BUSCO genes themselves could be duplicated in the *E. pauciflora* genome. Taken together, these four metrics suggest that the MaSuRCA_35kb assembly is the most complete, the most contiguous, and the most accurate among the assemblies we produced.

The other three metrics assess the correctness of every assembly, and also suggest that the best assemblies for our data are produced by MaSuRCA (Table 3). The MaSuRCA assemblies (1kb and 35kb) had the lowest error rates (0.006 errors per base for short-read mapping and 0.166 for long-read mapping in both assemblies), and the smallest total number of structural variants estimated from the long validation reads (4,017 structural variants for the MaSuRCA_35kb assembly). Flye and SMARTdenovo assemblies tended to perform the worst on these metrics, although we note that these results will be affected by the fact that the MaSuRCA assemblies contain more

duplicated genome regions (see above), which will tend to reduce the estimated error rates and number of structural variants, because duplicated regions can accurately represent heterozygous variants that will be present in the reads. CGAL ranked MaSuRCA assemblies as the best (1kb likelihood: -1,774,303 and 35kb likelihood: -1,790,386) as the best, and the SMARTdenovo_35kb assembly as the worst (likelihood: -5,869,476).

Finally, to further investigate the different assemblies, we compared the genome sequence similarity between different assemblies using NUCmer module of MUMmer (Fig. 4), with the minimum identity set to 75. Notably, around 8% of the sequence of Canu/SMARTdenovo/Flye/MaSuRCA assemblies failed to align to Marvel_35kb assembly (Fig. 4), which, along with the low genome completeness (BUSCO scores) of the Marvel_35kb assembly (Table 3), suggest that the Marvel_35kb assembly may contain many more small duplicated regions than other assemblies. In turn, these duplicated regions may explain the fact that Marvel_35kb assembly has the lowest genome completeness but not the smallest genome size compared to other assemblies (Table 3). Other assemblies have rough 97% - 99% of similarity to each other.

Based on the eight metrics we used above (Table 3), we suggest that the MaSuRCA_35kb assembly represents the most accurate representation of the *E. pauciflora* genome. We note, though, that the Flye assembler only took 1-3% of runtime of the other assemblers used in this paper (Table 1), and produced genome assemblies

that were of similar quality to the MaSuRCA_35kb assembly in many respects. The Marvel_35kb assembly received the worst scores on many metrics, and also appears to be missing roughly ~10% of the genome according to BUSCO scores and genome sequence similarity analyses compared to other assemblies (Table 3).

Comparative genome analysis between *E. pauciflora* and *E. grandis*

Using the MaSuRCA_35kb assembly, we estimate that the *E. pauciflora* genome is 594,871,467 bp in length, with 416 contigs and a contig N50 of 3,235 kb. The genome has up to 0.006 errors per base. Around 94% of complete BUSCO genes were identified in this *E. pauciflora* genome assembly.

E. grandis is the only published *Eucalyptus* genome that is assembled to chromosome level. We therefore compared *E. grandis* with our *E. pauciflora* genome. The *E. grandis* contains 691.43 Mb of sequence, roughly 16% larger than the *E. pauciflora* genome. We compared these two genome assemblies using the NUCmer module of MUMmer to perform whole genome alignment as described above. This alignment shows that the *E. pauciflora* genome assembly covers just 61.56% of the *E. grandis* genome sequence, leaving approximately 265 Mb of the *E. grandis* genome sequence not covered by the *E. pauciflora* assembly, and 113 Mb of the *E. pauciflora* assembly not covered by the *E. grandis* assembly. Despite this, the coverage of the *E. pauciflora* assembly when mapped to the 11 chromosome-scale scaffolds of the *E. grandis* genome is fairly constant (Fig. 5A), suggesting either that many of these differences result from small

errors in both assemblies, and/or from relatively small-scale differences in the underlying genomes.

To examine whether the differences between *E. pauciflora* and *E. grandis* could be explained by their repeat content, we annotated repetitive elements of *E. pauciflora* and *E. grandis* with RepeatMasker v4.0.7 (RepeatMasker, RRID:SCR_012954) [62]. Although the repeats of *E. grandis* have been annotated before [16], we reannotated them here to make a direct comparison of the repeat content using an identical pipeline for both genomes. First, we created the custom consensus repeat library using RepeatModeler v1.0.11 (RepeatModeler, RRID:SCR_015027) [63] with parameter "-engine ncbi". The classifier was built upon Repbase v20170127 [64]. Then we merged the repeat libraries from RepeatModeler and LTR retrotransposon candidates from LTR retriever to create a comprehensive repeat library as the input for RepeatMasker. We ran the RepeatMasker with "-engine ncbi" model. We used the 'calcDivergenceFromAlign.pl" script in RepeatMasker pipeline to calculate the Kimura divergence values, and plotted the repeat landscape with repeats presented in both *E. pauciflora* and *E. grandis* genomes (Fig. 5B).

The repeat content of the two genomes is similar. The *E. pauciflora* genome contains 44.77% of repetitive elements, compared to 41.22% in *E. grandis*. Retrotransposons account for 29.53% of *E. pauciflora* genome, and 26.94% in *E. grandis*, and DNA transposons account for 6.04% and 4.80% of the genome in *E. pauciflora* and *E.*

grandis, respectively. Both of two genomes show roughly two waves of repeat expansion in the repeat landscapes, which is most likely explained by a shared inheritance of most of the repeats in the two genomes (Fig. 5B).

Conclusions

Here, we report a high-quality draft haploid genome of *E. pauciflora*. It is the first *Eucalyptus* genome assembled with third-generation sequencing reads (Nanopore sequencing), and is the third nuclear genome of *Eucalyptus* species. Due to the economic and ecological importance of *Eucalyptus*, this high-quality genome will support further analysis on *Eucalyptus* and its related species. Additionally, this study will provide useful information for *de novo* plant genome assembly with Nanopore sequencing reads. Finally, the approaches used in this study to assess and compare different assemblies should help in assessing and choosing among many potential genome assemblies.

Table 1. Raw (before polish and haplotig removal) assembly statistics.

	Long-read^	Short-read	Assembler	Assembly time (CPU hours)*	Length (bp)	contigs	Largest contig (bp)	N50 (bp)	L50	GC	Percent Ns
Canu_1kb	≥1 kb (~174x)	X	Canu	~300,000	871,577,052	2,867	7,123,373	629,835	259	39.18%	0.00%
Canu_35kb	≥35 kb (~40x)	X	Canu	~50,000	825,916,527	2,550	10,153,603	962,598	158	39.18%	0.00%
SMARTdenovo_1kb	≥1 kb (~174x)	X	SMARTdenovo	~8,000	610,858,639	729	6,287,341	1,711,661	107	39.29%	0.00%
SMARTdenovo_35kb	≥35 kb (~40x)	X	SMARTdenovo	~4,000	586,903,502	704	9,494,401	1,868,532	91	39.27%	0.00%
Flye_1kb	≥1 kb (~174x)	X	Flye	~700	596,007,484	5,930	2,755,662	255,434	652	39.12%	0.00%
Flye_35kb	≥35 kb (~40x)	X	Flye	~500	561,349,738	4,145	2,407,003	352,050	448	39.17%	0.00%
Marvel_35kb	≥35 kb (~40x)	X	Marvel	~28,000	649,061,435	1,181	6,453,759	795,971	182	39.07%	0.00%
MaSuRCA_1kb	≥1 kb (~174x)	~228x	MaSuRCA	~23,000	778,288,575	1,311	12,224,271	1,885,174	95	39.35%	0.04%
MaSuRCA_35kb	≥35 kb (~40x)	~228x	MaSuRCA	~21,000	773,035,614	1,703	8,684,546	1,304,720	146	39.39%	0.09%

^all long-reads were corrected by Canu before assembly. The Canu correction step took around 200,000 CPU hours, which has not been included in the assembly runtime.

*with around 1 Tb of RAM.

Table 2. Assembly size and assembly ploidy during polishing and haplotig removal.

	Stage 1	Assembly ploidy	Stage 2	Assembly ploidy	Stage 3	Assembly ploidy	Stage 4	Assembly ploidy	Stage 5	Assembly ploidy
Canu_1kb	871,577,052	1.74	893,781,515	1.79	645,703,255	1.29	622,473,836	1.24	622,218,742	1.24
Canu_35kb	825,916,527	1.65	847,395,928	1.69	605,520,689	1.21	586,032,599	1.17	585,785,283	1.17

SMARTdenovo_1kb	599,580,691	1.20	610,858,639	1.22	514,822,476	1.03	514,822,476	1.03	514,714,831	1.03
SMARTdenovo_35kb	575,805,356	1.15	586,903,502	1.17	504,644,753	1.01	504,644,753	1.01	504,515,539	1.01
Flye_1kb	596,007,484	1.19	593,219,654	1.19	529,107,244	1.06	528,619,533	1.06	528,563,896	1.06
Flye_35kb	561,349,738	1.12	561,597,192	1.12	517,329,093	1.03	517,061,277	1.03	516,992,152	1.03
Marvel_35kb	649,061,435	1.30	666,317,308	1.33	547,630,224	1.10	537,813,575	1.08	537,615,613	1.08
MaSuRCA_1kb	778,288,575	1.56	778,307,850	1.56	608,764,671	1.22	594,680,200	1.19	594,528,099	1.19
MaSuRCA_35kb	773,035,614	1.55	773,071,231	1.55	608,629,204	1.22	595,020,257	1.19	594,871,467	1.19

Stage 1: Raw Assembly size (bp) before polishing. Stage 2: Assembly size (bp) after polishing. Stage 3: Assembly size (bp) after purge Haplotigs. Stage 4: Assembly size (bp) after Purge Haplotigs an GCICA (bp). Stage 5: Assembly size (bp) after Purge Haplotigs and GCICA and extra polishing.

Table 3. The comparison of final assemblies.

	Length (bp)	Contig number	Contig N50 (bp)	BUSCO score (1440 genes in total)						LAI scores	Assembly ploidy	Short-read mapping		Long-read mapping		CGAL scores	Structural variants
				Complete genes		Duplicated genes		Fragmented genes				Mapping rate	Error rate	Mapping rate	Error rate		
Canu_1kb	622,218,742	895	1,502,325	1,346	93.47%	183	12.71%	23	1.60%	7.04	1.24	96.02%	0.0061	91.73%	0.1661	-1.959E+06	4,243
Canu_35kb	585,785,283	655	2,258,674	1,345	93.40%	138	9.58%	29	2.01%	5.34	1.17	95.52%	0.0066	92.64%	0.1677	-2.226E+06	5,043
SMARTdenovo_1kb	514,714,831	364	2,092,790	1,342	93.19%	100	6.94%	27	1.88%	7.02	1.03	**98.42%**	0.0080	92.38%	0.1678	-4.275E+06	5,940
SMARTdenovo_35kb	504,515,539	370	2,178,079	1,341	93.13%	100	6.94%	30	2.08%	6.73	**1.01**	98.35%	0.0082	92.20%	0.1679	-5.869E+06	6,024
Flye_1kb	528,563,896	2,947	295,613	1,344	93.33%	100	6.94%	31	2.15%	5.70	1.06	94.86%	0.0077	**93.04%**	0.1694	-2.536E+06	7,137
Flye_35kb	516,992,152	2,548	385,290	1,336	92.78%	**90**	**6.25%**	31	2.15%	6.50	1.03	94.24%	0.0080	92.34%	0.1699	-2.726E+06	7,458
Marvel_35kb	537,615,613	730	1,202,845	1,180	81.94%	153	10.63%	32	2.22%	3.77	1.08	87.37%	0.0075	85.18%	0.1689	-4.451E+06	5,162
MaSuRCA_1kb	594,528,099	415	3,234,447	**1,362**	**94.58%**	201	13.96%	**21**	**1.46%**	9.27	1.19	94.91%	**0.0060**	91.57%	0.1656	**-1.774E+06**	4,020
MaSuRCA_35kb	594,871,467	416	**3,234,549**	**1,362**	**94.58%**	200	13.89%	**21**	**1.46%**	**9.31**	1.19	94.92%	**0.0060**	91.49%	**0.1655**	-1.790E+06	**4,017**

Note: The best value of each assessment is highlighted in bold.

559

Availability of supporting data

The *E. pauciflora* genome project was deposited at NCBI under BioProject number PRJNA450887. The whole genome sequencing data are available in the Sequence Read Archive with accession number SRR7153044-SRR7153116. The scripts we used in this paper, including the genome assembly, genome polishing, repeat annotation and genome assessments are available in the Github (https://github.com/asdcid/Eucalyptus-pauciflora-genome-assembly). Also, a single universal pipeline contained the assessment methods we used in this paper is available on Github at https://github.com/asdcid/Genome_Assembly_Assessment.

Additional files

Additional file 1: A png format with Fig. S1 (GenomeScope result of *E. pauciflora*.)

Additional file 2: A png format with Fig. S2 (Genome contamination detection. Almost all sequences were matched the sequences in streptophyta phylum group. No contamination was found.)

Additional file 3: A word format with Supplementary result (Supplementary result.)

Additional file 4: A xlsx format with Table S1 (The comparison of assemblies with corrected and uncorrected long-read datasets.)

Additional file 5: A xlsx format with Table S2 (The comparison of polishing results of raw assemblies.)

Additional file 6: A xlsx format with Table S3 (The comparison of polishing result of each genome after haplotig removal.)

Abbreviations

BUSCO: Benchmarking Universal Single-Copy Orthologs; CGAL: computing genome assembly likelihoods; *Eucalyptus grandis*: *E. grandis*; *Eucalyptus pauciflora*: *E. pauciflora*; the National Center for Biotechnology Information: NCBI; long-terminal repeat: LTR; long-terminal repeat assembly index: LAI.

Conflict of Interest

The authors declare that they have no competing financial interests.

Ethics Statement

E. pauciflora leaves were collected from a single *E. pauciflora* tree in Thredbo, Kosciuszko National Park, New South Wales, Australia (Latitude −36.49433, Longitude 148.282983). The written permission was from the Scientific Licensing office of the Office of Environment and Heritage for New South Wales: www.licence.nsw.gov.au, in accordance with national guidelines in Australia. Tissues were not deposited as voucher specimens.

Funding

This research is supported by the Australian Research Council Future Fellowship, FT140100843 to Rob Lanfear and FT180100024 to Benjamin Schwessinger.

Author Contributions

AD, DK, RL and WW conceived this project. AMS and RL performed sample collection for Illumina sequencing. AMS extracted genomic DNA, and constructed library for Illumina sequencing. RL and MS carried out sample collection for Nanopore sequencing. MS and BS performed DNA extraction, library preparation, and Nanopore sequencing. DK performed long-read polishing and Canu 1kb assembly, whereas AD performed Canu_35kb, Flye_1kb Flye_35kb and Marvel_35kb assemblies and contamination detection. AD and WW conducted the whole genome alignment analysis. WW conducted all the remaining analyses. AD, BS, DK, RL and WW were involved in data interpretation. AD, RL and WW drafted the original manuscript. RL and WW finalized the manuscript. All authors read and approved the final manuscript.

References

1. Department of Agriculture and Water Resources. Australian forest profiles Eucalypt. 2016.
2. Williams JE. Biogeographic Patterns of Three Sub-Alpine Eucalypts in South-East Australia with Special Reference to Eucalyptus pauciflora Sieb. Ex Spreng. Journal of Biogeography. 1991;18 2:223-30.
3. Boland DJ, Brooker MIH, Chippendale GM, Hall N, Hyland BPM, R.D. J, et al. Forest

trees of Australia. CSIRO, Canberra. 2002.

4. Gauli A, Vaillancourt RE, Bailey TG, Steane DA and Potts BM. Evidence for local climate adaptation in early-life traits of Tasmanian populations of Eucalyptus pauciflora. Tree Genetics & Genomes. 2015;11:104-15.

5. Cochrane PM and Slatyer RO. Water relations of Eucalyptus pauciflora near the alpine tree line in winter. Tree Physiol. 1988;4 1:45-52.

6. Evans JR and Vogelmann TC. Photosynthesis within isobilateral Eucalyptus pauciflora leaves. New Phytol. 2006;171 4:771-82. doi:10.1111/j.1469-8137.2006.01789.x.

7. Warren CR. Uptake of inorganic and amino acid nitrogen from soil by Eucalyptus regnans and Eucalyptus pauciflora seedlings. Tree Physiol. 2009;29 3:401-9. doi:10.1093/treephys/tpn037.

8. Buckley TN, Turnbull TL, Pfautsch S and Adams MA. Nocturnal water loss in mature subalpine Eucalyptus delegatensis tall open forests and adjacent E. pauciflora woodlands. Ecol Evol. 2011;1 3:435-50. doi:10.1002/ece3.44.

9. Martorell S, Diaz-Espejo A, Medrano H, Ball MC and Choat B. Rapid hydraulic recovery in Eucalyptus pauciflora after drought: linkages between stem hydraulics and leaf gas exchange. Plant Cell Environ. 2014;37 3:617-26. doi:10.1111/pce.12182.

10. Way DA, Holly C, Bruhn D, Ball MC and Atkin OK. Diurnal and seasonal variation in light and dark respiration in field-grown Eucalyptus pauciflora. Tree Physiol. 2015;35 8:840-9. doi:10.1093/treephys/tpv065.

11. Prior LD, Paul KI, Davidson NJ, Hovenden MJ, Nichols SC and Bowman DJMS. Evaluating carbon storage in restoration plantings in the Tasmanian Midlands, a highly

modified agricultural landscape. The Rangeland Journal. 2015;37 5:477-88. doi:https://doi.org/10.1071/RJ15070.

12. Wang W, Schalamun M, Morales-Suarez A, Kainer D, Schwessinger B and Lanfear R. Assembly of chloroplast genomes with long- and short-read data: a comparison of approaches using Eucalyptus pauciflora as a test case. BMC Genomics. 2018;19 1:977. doi:10.1186/s12864-018-5348-8.

13. Gauli A, Vaillancourt RE, Steane DA, Bailey TG and Potts BM. Effect of forest fragmentation and altitude on the mating system of Eucalyptus pauciflora (Myrtaceae). Australian Journal of Botany. 2014;61 8:622-32. doi:https://doi.org/10.1071/BT13259.

14. Gauli A, Steane DA, Vaillancourt RE and Potts BM. Molecular genetic diversity and population structure in <i>Eucalyptus pauciflora</i> subsp. <i>pauciflora</i> (Myrtaceae) on the island of Tasmania. Australian Journal of Botany. 2014;62 3:175-88. doi:https://doi.org/10.1071/BT14036.

15. Thornhill AH, Crisp MD, Külheim C, Lam KE, Nelson LA, Yeates DK, et al. A dated molecular perspective of eucalypt taxonomy, evolution and diversification. Australian Systematic Botany. 2019;32 1:29-48. doi:https://doi.org/10.1071/SB18015.

16. Myburg AA, Grattapaglia D, Tuskan GA, Hellsten U, Hayes RD, Grimwood J, et al. The genome of Eucalyptus grandis. Nature. 2014;510 7505:356-62. doi:10.1038/nature13308.

17. Hirakawa H, Nakamura Y, Kaneko T, Isobe S, Sakai H, Kato T, et al. Survey of the genetic information carried in the genome of Eucalyptus camaldulensis. Plant Biotechnology. 2011;28 5:471-80. doi:10.5511/plantbiotechnology.11.1027b.

18. Simao FA, Waterhouse RM, Ioannidis P, Kriventseva EV and Zdobnov EM. BUSCO: assessing genome assembly and annotation completeness with single-copy orthologs. Bioinformatics. 2015;31 19:3210-2. doi:10.1093/bioinformatics/btv351.

19. Gurevich A, Saveliev V, Vyahhi N and Tesler G. QUAST: quality assessment tool for genome assemblies. Bioinformatics. 2013;29 8:1072-5. doi:10.1093/bioinformatics/btt086.

20. Rahman A and Pachter L. CGAL: computing genome assembly likelihoods. Genome Biol. 2013;14 1:R8. doi:10.1186/gb-2013-14-1-r8.

21. Ou S, Chen J and Jiang N. Assessing genome assembly quality using the LTR Assembly Index (LAI). Nucleic Acids Research. 2018:gky730-gky. doi:10.1093/nar/gky730.

22. Slovin JP, Schmitt K and Folta KM. An inbred line of the diploid strawberry Fragaria vesca f. semperflorens for genomic and molecular genetic studies in the Rosaceae. Plant Methods. 2009;5:15. doi:10.1186/1746-4811-5-15.

23. Yasui Y, Hirakawa H, Oikawa T, Toyoshima M, Matsuzaki C, Ueno M, et al. Draft genome sequence of an inbred line of Chenopodium quinoa, an allotetraploid crop with great environmental adaptability and outstanding nutritional properties. DNA Res. 2016;23 6:535-46. doi:10.1093/dnares/dsw037.

24. Arumugasundaram S, Ghosh M, Veerasamy S and Ramasamy Y. Species Discrimination, Population Structure and Linkage Disequilibrium in Eucalyptus camaldulensis and Eucalyptus tereticornis Using SSR Markers. PLOS ONE. 2011;6 12:e28252. doi:10.1371/journal.pone.0028252.

25. Chin CS, Peluso P, Sedlazeck FJ, Nattestad M, Concepcion GT, Clum A, et al. Phased diploid genome assembly with single-molecule real-time sequencing. Nat Methods. 2016;13 12:1050-4. doi:10.1038/nmeth.4035.

26. Garg S, Rautiainen M, Novak AM, Garrison E, Durbin R and Marschall T. A graph-based approach to diploid genome assembly. Bioinformatics. 2018;34 13:i105-i14. doi:10.1093/bioinformatics/bty279.

27. Pryszcz LP, Németh T, Gácser A and Gabaldón T. Genome Comparison of Candida orthopsilosis Clinical Strains Reveals the Existence of Hybrids between Two Distinct Subspecies. Genome Biology and Evolution. 2014;6 5:1069-78. doi:10.1093/gbe/evu082.

28. Roach MJ, Schmidt SA and Borneman AR. Purge Haplotigs: Synteny Reduction for Third-gen Diploid Genome Assemblies. bioRxiv. 2018; doi:10.1101/286252.

29. Vezzi F, Narzisi G and Mishra B. Reevaluating assembly evaluations with feature response curves: GAGE and assemblathons. PLoS One. 2012;7 12:e52210. doi:10.1371/journal.pone.0052210.

30. Hunt M, Kikuchi T, Sanders M, Newbold C, Berriman M and Otto TD. REAPR: a universal tool for genome assembly evaluation. Genome Biol. 2013;14 5:R47. doi:10.1186/gb-2013-14-5-r47.

31. Schmidt MH, Vogel A, Denton AK, Istace B, Wormit A, van de Geest H, et al. De Novo Assembly of a New Solanum pennellii Accession Using Nanopore Sequencing. Plant Cell. 2017;29 10:2336-48. doi:10.1105/tpc.17.00521.

32. Costa MD, Artur MA, Maia J, Jonkheer E, Derks MF, Nijveen H, et al. A footprint of

desiccation tolerance in the genome of Xerophyta viscosa. Nat Plants. 2017;3:17038. doi:10.1038/nplants.2017.38.

33. Schirmer M, D'Amore R, Ijaz UZ, Hall N and Quince C. Illumina error profiles: resolving fine-scale variation in metagenomic sequencing data. BMC Bioinformatics. 2016;17:125. doi:10.1186/s12859-016-0976-y.

34. Istace B, Friedrich A, d'Agata L, Faye S, Payen E, Beluche O, et al. de novo assembly and population genomic survey of natural yeast isolates with the Oxford Nanopore MinION sequencer. Gigascience. 2017;6 2:1-13. doi:10.1093/gigascience/giw018.

35. Giordano F, Aigrain L, Quail MA, Coupland P, Bonfield JK, Davies RM, et al. De novo yeast genome assemblies from MinION, PacBio and MiSeq platforms. Sci Rep. 2017;7 1:3935. doi:10.1038/s41598-017-03996-z.

36. Koren S, Walenz BP, Berlin K, Miller JR, Bergman NH and Phillippy AM. Canu: scalable and accurate long-read assembly via adaptive k-mer weighting and repeat separation. Genome Res. 2017;27 5:722-36. doi:10.1101/gr.215087.116.

37. Ruan J. Ultra-fast de novo assembler using long noisy reads. https://github.com/ruanjue/smartdenovo (2016). Accessed Sept 2019.

38. Kolmogorov M, Yuan J, Lin Y and Pevzner PA. Assembly of long, error-prone reads using repeat graphs. Nature Biotechnology. 2019; doi:10.1038/s41587-019-0072-8.

39. Nowoshilow S, Schloissnig S, Fei JF, Dahl A, Pang AWC, Pippel M, et al. The axolotl genome and the evolution of key tissue formation regulators. Nature. 2018;554 7690:50-5. doi:10.1038/nature25458.

40. Zimin AV, Marcais G, Puiu D, Roberts M, Salzberg SL and Yorke JA. The MaSuRCA

genome assembler. Bioinformatics. 2013;29 21:2669-77. doi:10.1093/bioinformatics/btt476.

41. Schalamun M and Schwessinger B. High molecular weight gDNA extraction after Mayjonade et al. optimised for eucalyptus for nanopore sequencing. Protocolsio 2017. doi:dx.doi.org/10.17504/protocols.io.ka2csge.

42. Wick RR. Porechop. https://github.com/rrwick/Porechop. Accessed 13 Jul 2017.

43. De Coster W, D'Hert S, Schultz DT, Cruts M and Van Broeckhoven C. NanoPack: visualizing and processing long-read sequencing data. Bioinformatics. 2018;34 15:2666-9. doi:10.1093/bioinformatics/bty149.

44. Suarez AM and Rutherford S. gDNA Extraction of Eucalypts pauciflora for full genome sequencing. Protocolsio. 2018. doi:dx.doi.org/10.17504/protocols.io.j7ecrje.

45. BBMap. http://sourceforge.net/projects/bbmap/. Accessed 16 Jun 2017.

46. Vurture GW, Sedlazeck FJ, Nattestad M, Underwood CJ, Fang H, Gurtowski J, et al. GenomeScope: fast reference-free genome profiling from short reads. Bioinformatics. 2017;33 14:2202-4. doi:10.1093/bioinformatics/btx153.

47. Simpson JT and Durbin R. Efficient de novo assembly of large genomes using compressed data structures. Genome Res. 2012;22 3:549-56. doi:10.1101/gr.126953.111.

48. Marcais G and Kingsford C. A fast, lock-free approach for efficient parallel counting of occurrences of k-mers. Bioinformatics. 2011;27 6:764-70. doi:10.1093/bioinformatics/btr011.

49. Edwards RJ, Tuipulotu DE, Amos TG, O'Meally D, Richardson MF, Russell TL, et al.

Draft genome assembly of the invasive cane toad, Rhinella marina. Gigascience. 2018; doi:10.1093/gigascience/giy095.

50. Wang W and Lanfear R. SplitReads. https://github.com/roblanf/splitreads. Accessed 13 Oct 2017 2018.

51. Ou S and Jiang N. LTR_retriever: A Highly Accurate and Sensitive Program for Identification of Long Terminal Repeat Retrotransposons. Plant Physiol. 2018;176 2:1410-22. doi:10.1104/pp.17.01310.

52. Sedlazeck FJ, Rescheneder P, Smolka M, Fang H, Nattestad M, von Haeseler A, et al. Accurate detection of complex structural variations using single-molecule sequencing. Nat Methods. 2018;15 6:461-8. doi:10.1038/s41592-018-0001-7.

53. Langmead B and Salzberg SL. Fast gapped-read alignment with Bowtie 2. Nat Methods. 2012;9 4:357-9. doi:10.1038/nmeth.1923.

54. Okonechnikov K, Conesa A and Garcia-Alcalde F. Qualimap 2: advanced multi-sample quality control for high-throughput sequencing data. Bioinformatics. 2016;32 2:292-4. doi:10.1093/bioinformatics/btv566.

55. Marcais G, Delcher AL, Phillippy AM, Coston R, Salzberg SL and Zimin A. MUMmer4: A fast and versatile genome alignment system. PLoS Comput Biol. 2018;14 1:e1005944. doi:10.1371/journal.pcbi.1005944.

56. Ruan J and Li H. Fast and accurate long-read assembly with wtdbg2. bioRxiv. 2019; doi:10.1101/530972.

57. Camacho C, Coulouris G, Avagyan V, Ma N, Papadopoulos J, Bealer K, et al. BLAST+: architecture and applications. BMC Bioinformatics. 2009;10:421. doi:10.1186/1471-

2105-10-421.

58. Laetsch D and Blaxter M. BlobTools: Interrogation of genome assemblies [version 1; referees: 2 approved with reservations]. F1000Research. 2017;6 1287 doi:10.12688/f1000research.12232.1.

59. Vaser R, Sovic I, Nagarajan N and Sikic M. Fast and accurate de novo genome assembly from long uncorrected reads. Genome Res. 2017;27 5:737-46. doi:10.1101/gr.214270.116.

60. Walker BJ, Abeel T, Shea T, Priest M, Abouelliel A, Sakthikumar S, et al. Pilon: an integrated tool for comprehensive microbial variant detection and genome assembly improvement. PLoS One. 2014;9 11 doi:10.1371/journal.pone.0112963.

61. W.Wang. Gene conservation informed contig alignment. https://github.com/asdcid/Gene-conservation-informed-contig-alignment (2018). Accessed 30 Oct 2018.

62. Smit A, Hubley R and Green P. RepeatMasker Open-4.0. http://wwwrepeatmaskerorg. 2015.

63. Smit A and Hubley R. RepeatModeler Open-1.0. http://wwwrepeatmaskerorg. 2015.

64. Bao W, Kojima KK and Kohany O. Repbase Update, a database of repetitive elements in eukaryotic genomes. Mob DNA. 2015;6:11. doi:10.1186/s13100-015-0041-9.

Figure legends

Figure 1: The *E. pauciflora* sequenced in this study. This *E. pauciflora* is located in

Thredbo, Kosciuszko National Park, New South Wales, Australia (36° 29′ 39.58″ N, 148° 16′ 58.73″ E).

Figure 2: **A.** The length of primary contigs and haplotigs between different assemblies. **B.** The comparison of complete BUSCO genes (1440 in total) between different primary contigs. **C.** The comparison of duplicated BUSCO genes between different primary contigs.

Figure 3: Structural variation analysis of different assembly primary contigs. Each variant was supported by at least 10 long-reads. **A.** The total event of each structural variances of each assembly. **B.** The insertion event of each assembly. **C.** The translocation event of each assembly. **D.** The Deletion event of each assembly.

Figure 4: The sequence coverage of whole genome alignment among different assemblies. The sequence coverage was calculated by the length of aligned reference sequence / the total length of reference genome.

Figure 5: **A.** The histogram of location and coverage of *E. pauciflora* genome aligned to the 11 chromosomes of *E. grandis*. The scale of y-axis is 0x-2x of coverage. Every bar is 1 Mb. The coverage was calculated by the total aligned length of *E. grandis* in each bar / the length of bar. If a site in *E. grandis* is aligned by *E. pauciflora* twice or more, this site will be counted twice or more. **B.** Repeat landscape comparison between *E. pauciflora* and *E. grandis*. Only repeats that are found in both genomes are shown. Older repeat insertions could accumulate more mutations compared to new repeat insertions. This leads to older repeat insertions to have accumulated a higher level of divergence (shown on the right size of the graph).

Fig 1. The E. pauciflora sequenced in this study

Click here to download Figure Fig_1.jpg

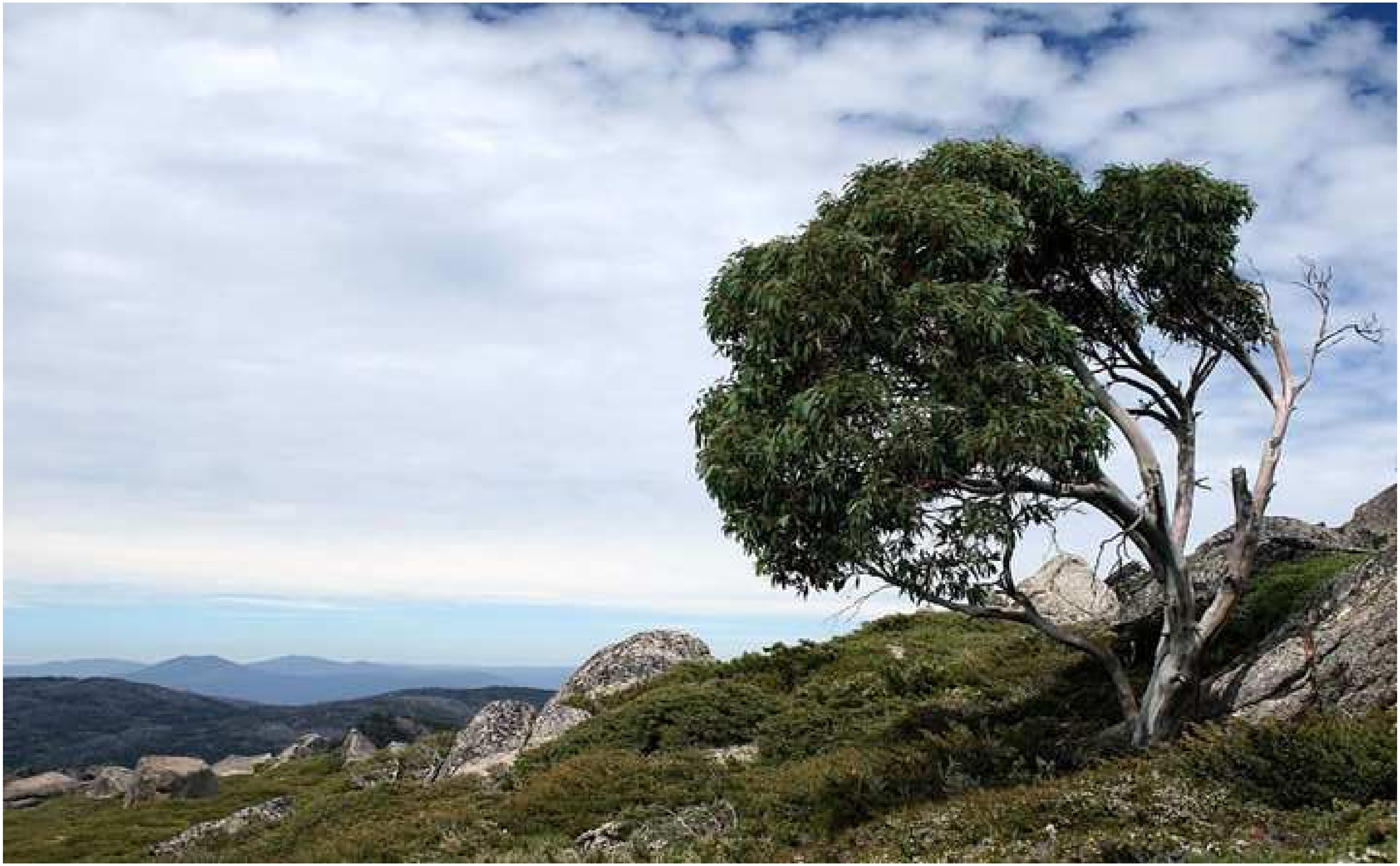

Fig 2. The length and BUSCO scores of primary contigs and haplotigs between different assemblies

Click here to download Figure Fig_2.png

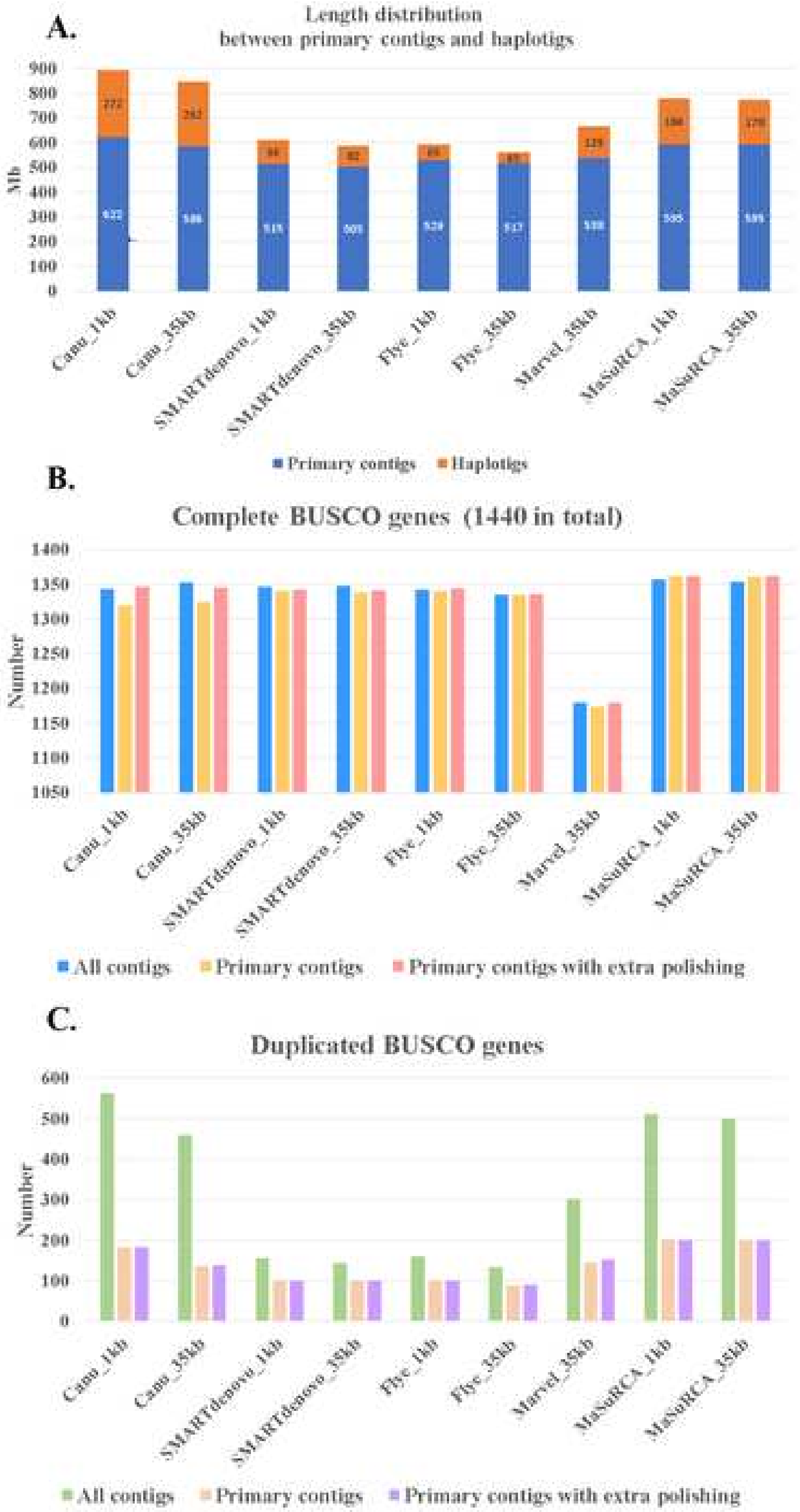

Fig 3. Structural variation analysis of different assembly primary contigs

Click here to download Figure Fig_3.png

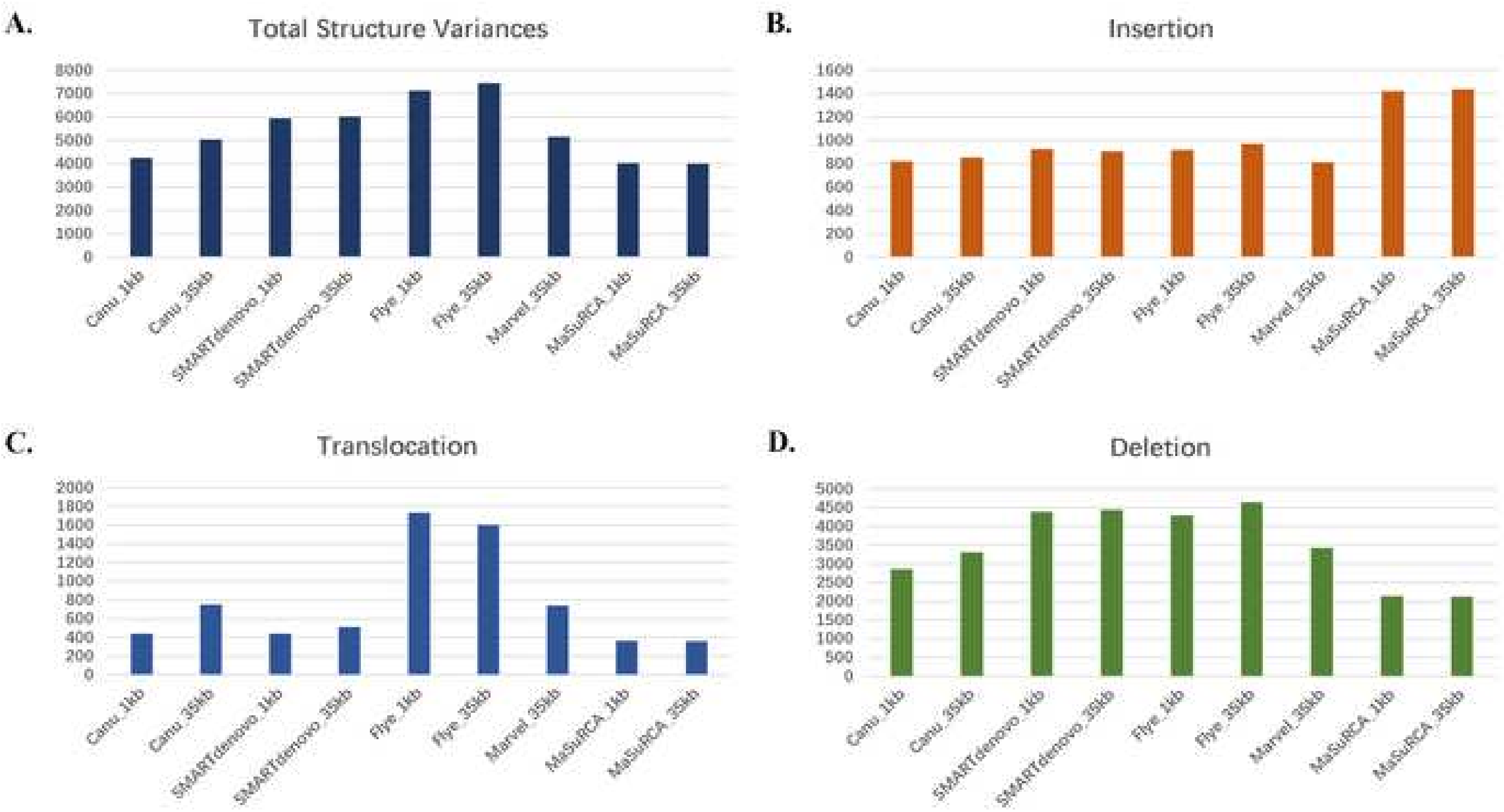

Fig 4. The sequence coverage of whole genome alignment among different assemblies

Click here to download Figure Fig_4.png

Reference \ Query	Canu_1kb	Canu_35kb	SMARTdenovo_1kb	SMARTdenovo_35kb	Flye_1kb	Flye_35kb	Marvel_35kb	MaSuRCA_1kb	MaSuRCA_35kb
Canu_1kb		99.37%	98.94%	98.94%	98.82%	98.83%	98.93%	99.12%	99.12%
Canu_35kb	99.24%		98.86%	98.89%	98.72%	98.74%	98.85%	99.03%	99.03%
SMARTdenovo_1kb	97.77%	97.77%		98.54%	97.58%	97.60%	97.48%	97.85%	97.85%
SMARTdenovo_35kb	97.73%	97.76%	98.42%		97.56%	97.58%	97.54%	97.85%	97.85%
Flye_1kb	99.14%	99.14%	99.08%	99.11%		99.24%	99.01%	99.18%	99.18%
flye_35kb	98.96%	99.02%	98.94%	99.00%	99.07%		98.84%	99.04%	99.04%
Marvel_35kb	91.77%	91.83%	91.11%	91.11%	91.12%	91.14%		91.94%	91.94%
MaSuRCA_1kb	98.72%	98.74%	98.60%	98.63%	98.42%	98.47%	98.59%		99.99%
MaSuRCA_35kb	98.73%	98.75%	98.62%	98.64%	98.43%	98.48%	98.60%	99.99%	

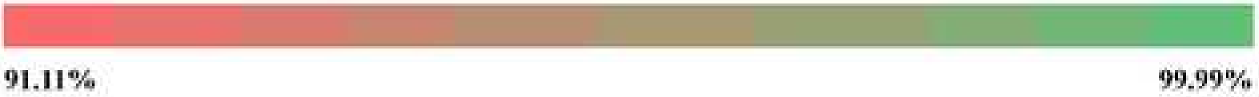

Fig 5. Whole genome alignment and repeat landscape comparison between E. pauciflora and E. grandis

Click here to download Figure Fig_5.png

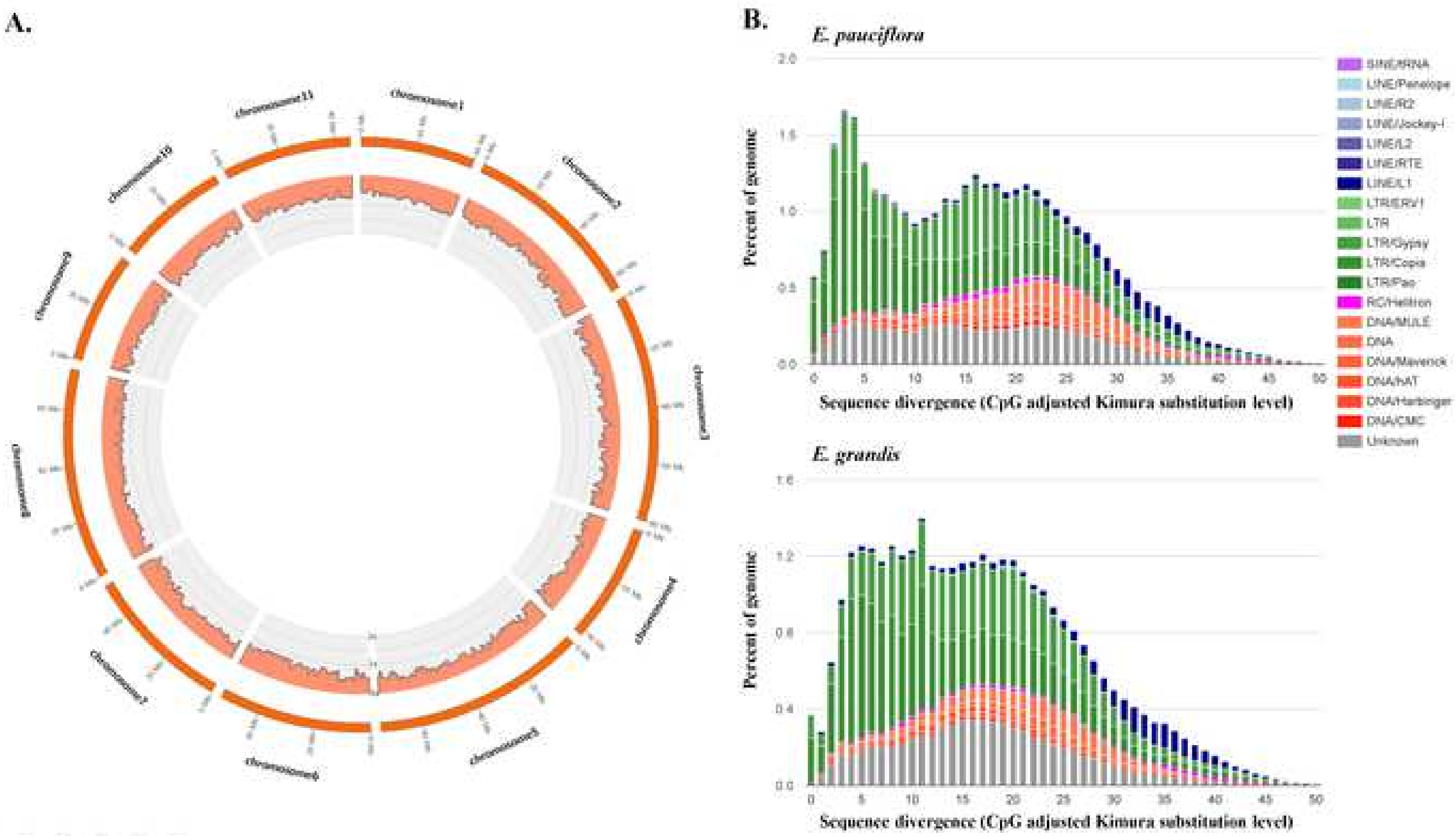

Fig S1. GenomeScope result of E. pauciflora

Click here to access/download
Supplementary Material
Fig_S1_GenomeScope result of E. pauciflora.png

Fig S2. Genome contamination detection

Click here to access/download
Supplementary Material
Fig_S2.png

Supplementary result

Click here to access/download
Supplementary Material
Supplementary result.docx

Table S1. The comparison of assemblies with corrected and uncorrected long-read datasets

Click here to access/download
Supplementary Material
Table_S1.xlsx

Table S2. The comparison of polishing results of raw assemblies

Click here to access/download
Supplementary Material
Table_S2.xlsx

Table S3. The comparison of polishing result of each genome after haplotig removal

Click here to access/download
Supplementary Material
Table_S3.xlsx

Response to reviewers

Click here to access/download
Supplementary Material
response to reviewers.pdf
