# Supplementary material for: The draft nuclear genome assembly of Eucalyptus pauciflora: a pipeline for comparing de novo assemblies
Source: Gigascience. 2020 Jan 2;9(1):giz160. doi: 10.1093/gigascience/giz160 (PMC6939829; doi:10.1093/gigascience/giz160)
Supplement: giz160_Supplemental_Figures_and_Tables [file giz160_supplemental_figures_and_tables.zip › Supplementary result.docx]

To quickly evaluate the performance of assemblies using corrected reads and uncorrected reads, we assembled the *E. pauciflora* genome with two efficient long-read only assemblers, Flye v2.3.5 [1] and wtdbg2 v2.5 [2]. The long-reads were corrected by Canu v1.6 [3] with default parameters except for setting corMinCoverage to 8. We then performed four assemblies with each assembler, using corrected 1 kb and 35 kb long-read datasets and uncorrected 1 kb and 35 kb long-read datasets, respectively (Table S1). All eight assemblies were polished with Pilon v1.22 (Pilon, RRID:SCR_014731) [4] using Bowtie2 with the short-read assembly dataset. We then used Purge Haplotigs [5] to separate the possible haplotigs, and assessed the contig N50, BUSCO scores and base-level error rate of each assembly (Addition file 4: Table S1) (details as described in the “Assembly ploidy and haplotig removal” and “Assessment of assembly quality with eight measures” sections of main text).

In general, assemblies with corrected reads have lower error rates and higher BUSCO scores compared to assemblies with uncorrected reads. For the contig N50, assemblies with corrected and uncorrected reads performed similarly with Flye assembler, but assemblies with uncorrected reads have 2-3 times longer N50 compared to assemblies with corrected reads in wtdbg2 assembler. In addition, Flye assemblies have better BUSCO scores and base-level errors than wtdbg2 assemblies. Therefore, we used the corrected long-read dataset in the further analysis.

**Reference**

1. Kolmogorov M, Yuan J, Lin Y and Pevzner PA. Assembly of long, error-prone reads using repeat graphs. Nature Biotechnology. 2019; doi:10.1038/s41587-019-0072-8.

2. Ruan J and Li H. Fast and accurate long-read assembly with wtdbg2. bioRxiv. 2019; doi:10.1101/530972.

3. Koren S, Walenz BP, Berlin K, Miller JR, Bergman NH and Phillippy AM. Canu: scalable and accurate long-read assembly via adaptive k-mer weighting and repeat separation. Genome Res. 2017;27 5:722-36. doi:10.1101/gr.215087.116.

4. Walker BJ, Abeel T, Shea T, Priest M, Abouelliel A, Sakthikumar S, et al. Pilon: an integrated tool for comprehensive microbial variant detection and genome assembly improvement. PLoS One. 2014;9 11 doi:10.1371/journal.pone.0112963.

5. Roach MJ, Schmidt SA and Borneman AR. Purge Haplotigs: Synteny Reduction for Third-gen Diploid Genome Assemblies. bioRxiv. 2018; doi:10.1101/286252.
