# Supplementary figures and images for: The draft nuclear genome assembly of Eucalyptus pauciflora: a pipeline for comparing de novo assemblies
Source: Gigascience. 2020 Jan 2;9(1):giz160. doi: 10.1093/gigascience/giz160 (PMC6939829; doi:10.1093/gigascience/giz160)

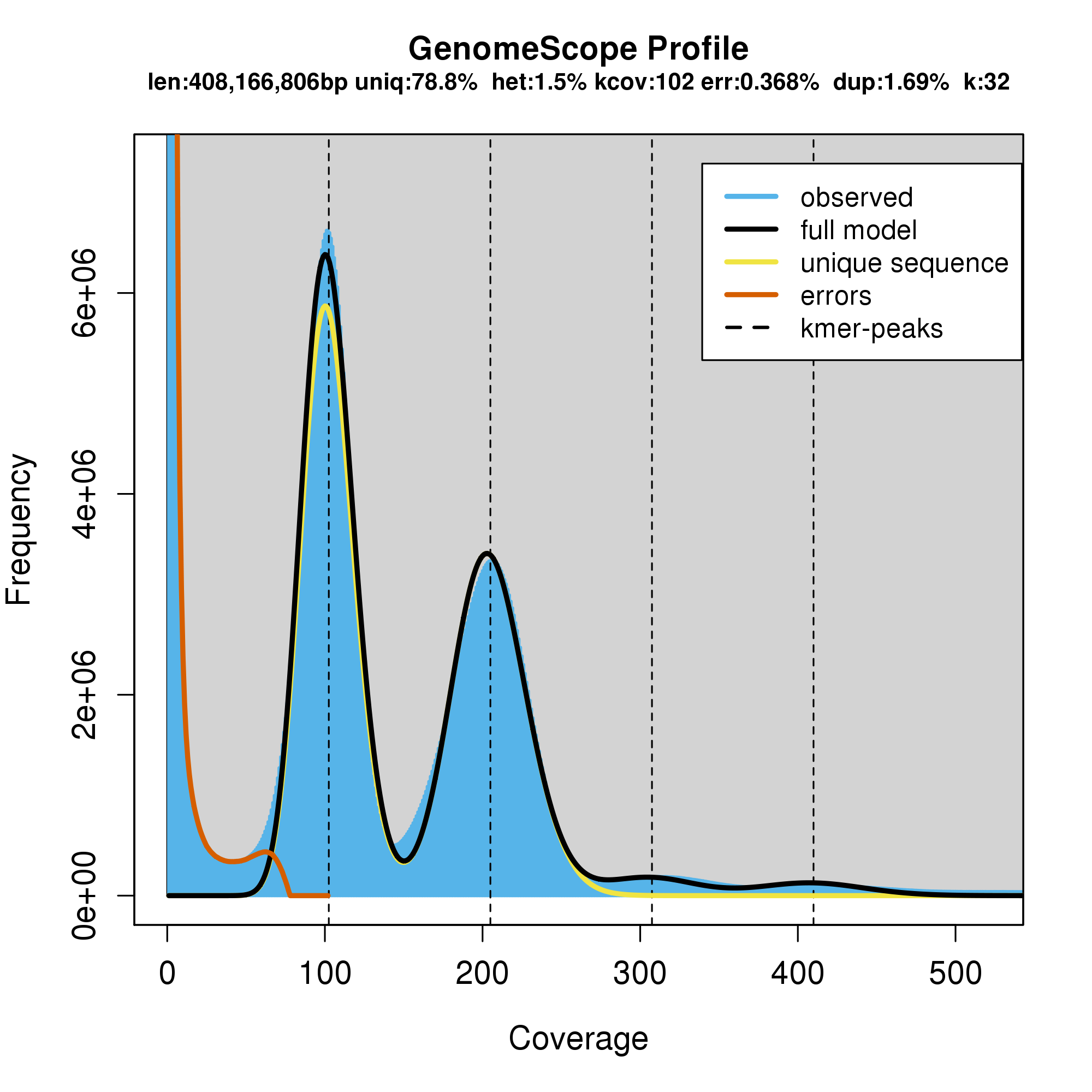

Supplement: giz160_Supplemental_Figures_and_Tables [file giz160_supplemental_figures_and_tables.zip › Fig_S1_GenomeScope result of E. pauciflora.png]

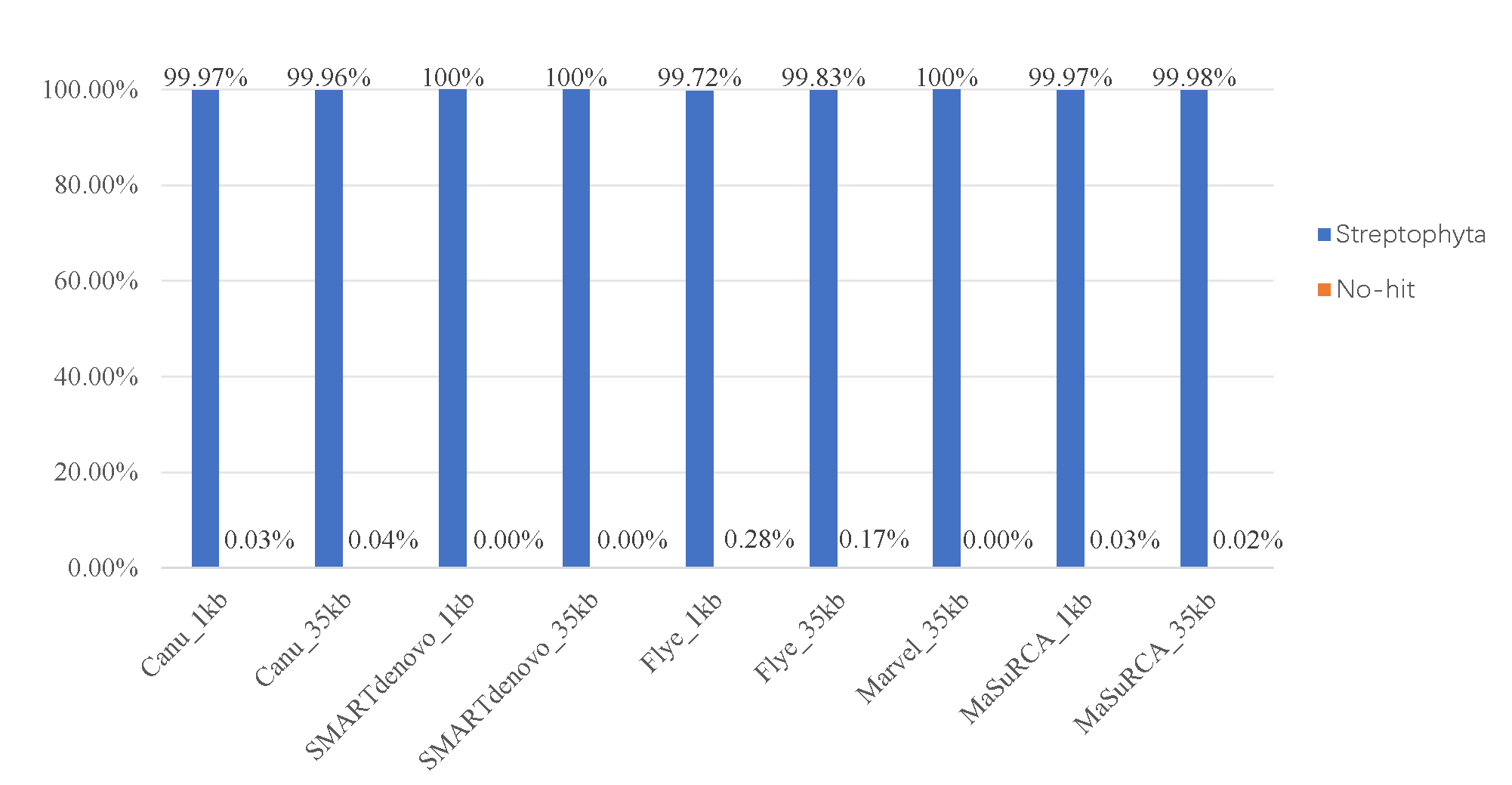

Supplement: giz160_Supplemental_Figures_and_Tables [file giz160_supplemental_figures_and_tables.zip › Fig_S2.png]
