# Supplementary material for: The draft nuclear genome assembly of Eucalyptus pauciflora: a pipeline for comparing de novo assemblies
Source: Gigascience. 2020 Jan 2;9(1):giz160. doi: 10.1093/gigascience/giz160 (PMC6939829; doi:10.1093/gigascience/giz160)
Supplement: giz160_Response_to_Reviewer_Comments_Original_Submission [file giz160_response_to_reviewer_comments_original_submission.pdf]

Author's Response To Reviewer Comments

Close

Dear Editor Zhou,
We would like to thank both you and the reviewers' comments. This is the point-by-point response to the comments.

Reviewer reports:
Reviewer #1: The authors had answered my questions point to point. In total, this revision make it much stronger to be published. However, I think the process of initial assembly seems a bit rough, e.g. there is no parameters tuning. I had downloaded the ONT data, and tried to assemble them using wtdbg2(v2.5), which would take little time to test. `wtdbg2 -x ont -g 0.5g -l 8192 ......` produced the best assembly in the aspect of contig statistic among all the assemblies in this paper, totaled in 510 Mb, N50 of 1.9 Mb and the longest contig was 12.6 Mb. So, I am worrying about whether the authors have tuned parameters carefully for other assemblers. If not, the conclusion "Additionally, this study will provide useful information for de novo plant genome assembly with Nanopore sequencing reads" is considered to be withdrawn.
A: Thanks for the comment. There are so many parameters for each assembler. As a user, not the assembler author, we are hard to understand the effect of each parameter, and we don't think we have enough time to test each of them. Therefore, we just tested a few different settings, rather than all. We have removed that conclusion as reviewer #1 suggested.

Reviewer #2: The author significantly improved their manuscript by carefully considered all reviewers comments. I am currently satisfied with the paper quality and would like to recommend it for publication in GigaScience.

I noticed a few minor issues in the paper that the authors may would like to take on board in the final version. Most of these issues were present in the original version of the manuscript as well but I have missed them in the first iteration due to many other misprints and problems.
* line 443: "SMARTdenovo assemblies (e.g.1.01 for the SMARTdenovo_35kb assembly vs. 1.19" --> missing space before 1.01
A: Thanks for the comment. We have added the space before 1.01.

* lines 466-7: "MaSuRCA assemblies as the best (1kb likelihood: -1,774,303 and 35kb likelihood: - 1,790,386) as the best" --> extra space between "-" and "1"; duplicated "as the best"
A: Thanks for the comment. We have deleted the duplicate.

* section "Abbreviations" (line 583): some abbreviations are given before their full forms (BUSCO, CGAL) while some others are stated after their full forms (E. grandis, E. pauciflora, NCBI, LTR, LAI). --> please unify this, I prefer the order used for BUSCO and CGAL
A: Thanks for the comment. We have fixed this mistake. Now all abbreviations are given before their full names.

* "References" (line 618):
#14 --> fix issues with italics special characters (like "< i >")
A: Thanks for the comment. We have fixed this problem now.

#28 --> this paper is published now https://bmcbioinformatics.biomedcentral.com/articles/10.1186/s12859-018-2485-7, please use this reference instead of bioRxiv preprint
A: Thanks for telling. We have changed the reference.

#50: Wang W and Lanfear R. SplitReads. https://github.com/roblanf/splitreads. Accessed 13 Oct 2017 2018. --> two years are specified
A: Thanks for the comment. We have deleted the 2017.

#62 and 63: --> fix the website address (wwwrepeatmaskerorg should include dots); also consider linking directly to the specific tools rather than the main page (e.g. http://www.repeatmasker.org/RepeatModeler/ for RepeatModeler); also consider updating the year for RepeatMasker, the latest version is from 2019.
A: Thanks for the comment. We have fixed this issue now. The year (2015) and the website of RepeatModeler (http://www.repeatmasker.org/) is the citation format suggested by RepeatMasker website, but we agree with you, your change makes the reference become better.

Best regards,
Weiwen Wang

Close
