# Supplementary material for: The draft nuclear genome assembly of Eucalyptus pauciflora: a pipeline for comparing de novo assemblies
Source: Gigascience. 2020 Jan 2;9(1):giz160. doi: 10.1093/gigascience/giz160 (PMC6939829; doi:10.1093/gigascience/giz160)
Supplement: giz160_Reviewer_2_Report_Original_Submission — Alexey Gurevich -- 11/15/2019 Reviewed [file giz160_reviewer_2_report_original_submission.pdf]

**Reviewer Report**

**Title: The draft nuclear genome assembly of Eucalyptus pauciflora: a pipeline for comparing de novo assemblies**

**Version: Original Submission** **Date:** 11/15/2019

**Reviewer name: Alexey Gurevich**

**Reviewer Comments to Author:**

The author significantly improved their manuscript by carefully considered all reviewers comments. I am currently satisfied with the paper quality and would like to recommend it for publication in GigaScience. I noticed a few minor issues in the paper that the authors may would like to take on board in the final version. Most of these issues were present in the original version of the manuscript as well but I have missed them in the first iteration due to many other misprints and problems.
* line 443: "SMARTdenovo assemblies (e.g.1.01 for the SMARTdenovo_35kb assembly vs. 1.19" --> missing space before 1.01
* lines 466-7: "MaSuRCA assemblies as the best (1kb likelihood: -1,774,303 and 35kb likelihood: - 1,790,386) as the best" --> extra space between "-" and "1"; duplicated "as the best"
* section "Abbreviations" (line 583): some abbreviations are given before their full forms (BUSCO, CGAL) while some others are stated after their full forms (E. grandis, E. pauciflora, NCBI, LTR, LAI). --> please unify this, I prefer the order used for BUSCO and CGAL
* "References" (line 618):
#14 --> fix issues with italics special characters (like "< i >")
#28 --> this paper is published now https://bmcbioinformatics.biomedcentral.com/articles/10.1186/s12859-018-2485-7, please use this reference instead of bioRxiv preprint
#50: Wang W and Lanfear R. SplitReads. https://github.com/roblanf/splitreads. Accessed 13 Oct 2017 2018. --> two years are specified
#62 and 63: --> fix the website address (wwwrepeatmaskerorg should include dots); also consider linking directly to the specific tools rather than the main page (e.g. http://www.repeatmasker.org/RepeatModeler/ for RepeatModeler); also consider updating the year for RepeatMasker, the latest version is from 2019.

**Level of Interest**

Please indicate how interesting you found the manuscript: Choose an item.

**Quality of Written English**

Please indicate the quality of language in the manuscript: Choose an item.

**Declaration of Competing Interests**

Please complete a declaration of competing interests, considering the following questions:

- Have you in the past five years received reimbursements, fees, funding, or salary from an organisation that may in any way gain or lose financially from the publication of this manuscript, either now or in the future?
- Do you hold any stocks or shares in an organisation that may in any way gain or lose financially from the publication of this manuscript, either now or in the future?
- Do you hold or are you currently applying for any patents relating to the content of the manuscript?
- Have you received reimbursements, fees, funding, or salary from an organization that holds or has applied for patents relating to the content of the manuscript?
- Do you have any other financial competing interests?
- Do you have any non-financial competing interests in relation to this paper?

If you can answer no to all of the above, write 'I declare that I have no competing interests' below. If your reply is yes to any, please give details below.

I declare that I have no competing interests

I agree to the open peer review policy of the journal. I understand that my name will be included on my report to the authors and, if the manuscript is accepted for publication, my named report including any attachments I upload will be posted on the website along with the authors' responses. I agree for my report to be made available under an Open Access Creative Commons CC-BY license (http://creativecommons.org/licenses/by/4.0/). I understand that any comments which I do not wish to be included in my named report can be included as confidential comments to the editors, which will not be published.

Choose an item.

To further support our reviewers, we have joined with Publons, where you can gain additional credit to further highlight your hard work (see: https://publons.com/journal/530/gigascience). On publication of this paper, your review will be automatically added to Publons, you can then choose whether or not to claim your Publons credit. I understand this statement.

Yes Choose an item.
